# Sustainable by design: a systematic review of factors for health promotion program sustainability

**DOI:** 10.1186/s12889-020-09091-9

**Published:** 2020-06-19

**Authors:** Andrea Bodkin, Shawn Hakimi

**Affiliations:** Public Health Ontario, 480 University Avenue, Suite 300, Toronto, ON M5G 1V2 Canada

**Keywords:** Program sustainability, Health promotion programs, Program planning, Public health, Routinization, Institutionalization

## Abstract

**Background:**

Sustaining health promotion programs (HPP) is critical to maintain their intended health benefits, community capacity, and to optimize resources and investment. However, not all programs are sustained beyond their initial implementation period. This is partly due to uncertainty regarding sustainability: lack of a clear definition; infrequent use of a sustainability framework; and lack of understanding of the factors that influence sustainability. The aim of this systematic review is to identify barriers and facilitators that promote or inhibit the sustainability of HPP, particularly those that can be considered in program planning.

**Methods:**

Two search strategies were used: 1) electronic database searching; and 2) grey literature searching. Inclusion criteria included papers published since 1998, in English, focusing on the sustainability of HPP that explicitly used a sustainability framework and specifically reported on facilitators and barriers to sustainability. Exclusion criteria included papers that addressed environmental, system or sector sustainability. Quality assessment was conducted on all included papers and a quality assessment tool was developed for grey literature. Data analysis included a thematic analysis, using an a *priori* framework to initially code barriers and facilitators, which were then grouped into factors for HPP sustainability. Factors were then analyzed for frequency, importance, and relevance, and categorized into one of three tiers.

**Results:**

Sixteen papers were included in this review. Eleven definitions of sustainability and 13 sustainability frameworks were used. A total of 83 barriers and 191 facilitators were identified and categorized into 14 factors: nine from the a *priori* framework, and five additional ones based on the results of our analysis. Tier 1 factors were the most important for sustainability with organizational capacity scoring the highest; tier 3, the least important.

**Conclusion:**

This review provides clarity regarding existing definitions of sustainability and sustainability frameworks. It identifies fourteen factors that influence program sustainability, which practitioners can consider when planning, developing and implementing HPP. In addition, it is important for practitioners to clearly articulate program elements that should be sustained, define sustainability as it relates to the context of their program, select a sustainability framework to guide their work, and consider these factors for sustainability.

## Background

The Ottawa Charter defines health promotion as the process of enabling people to increase control over their health, and its determinants, in order to improve health [1]. Health Promotion Programs (HPP) “improve population health outcomes by reducing preventable disease, injury or death, and taking action on health inequities” [2]. This highlights the vital role that HPP play in curbing health care costs in the current climate of scarce funding [3]. While resources for health promotion are limited, the expectation for HPP to generate results remains high [4]. As such, the health promotion field faces a predicament: to provide quality services that reduce disease burden and improve quality of life, while under budgetary constraints. This gives rise to the necessity for HPP to be sustained, so that health benefits do not end when the program does [5–7].

While sustainability is often thought of within the lens of funding; for instance, finding replacement funding when initial program funding comes to an end [8, 9], sustainability is more complex than this [10]. Program sustainability refers to the maintenance of health benefits, the continuation of a program within an organization, and capacity built in the recipient community to continue carrying out the program on its own [11]. Despite its importance, only 40 to 60% of HPP are sustained [6, 12], as project funding cycles are short and priorities shift over time [13]. Failure to sustain HPP can give rise to three “serious problems”:

1. The issue that a program was established to address remains or recurs.
2. Programs see their funding withdrawn before activities have been fully realized and outcomes reached, despite significant human, fiscal and technical start-up costs.
3. Diminished community support and lack of trust in communities with a history of programs that were abruptly/inappropriately terminated [11].

Several key challenges affect HPP sustainability. First, there is a lack of consistency regarding what ‘sustainability’ means and how it is defined [11, 14–17]. Second, many sustainability frameworks and tools exist, but few are validated [14]. Third, although it is often acknowledged that planning for sustainability should begin early in a program’s life cycle, [10, 11, 15, 16, 18–20] it tends to be a ‘latent’ concern often thought of at the end of the program when remaining staff, time and resources are limited [11].

In order to promote the sustainability of HPP, the factors that promote long term sustainability need to be understood [6, 14, 21]. This would allow for a clearer conceptual understanding of how a sustainable program is built from the outset.

This systematic review aims to identify the barriers and facilitators that influence the sustainability of HPP. Particular attention is given to those factors that can be considered during the program planning processes. Our research question is “what factors facilitate or influence the sustainability of health promotion programs?” In conducting this review, we assessed the body of literature on this topic to determine how sustainability is being defined, which frameworks are used to plan for sustainability and the overall implications for the health promotion and public health fields.

## Methods

We conducted a systematic review that focused on identifying barriers and facilitators for HPP sustainability. For this study, we used Grant and Booth’s 2009 definition of a systematic review: an evidence synthesis that adheres to guidelines on conduct of a review/method for integrating or comparing the findings from qualitative studies, looking for ‘themes’ or ‘constructs’ that lie in or across qualitative studies [22].

The authors (AB and SH) jointly developed a protocol for completing this review in advance, which included the following steps outlined in PRISMA guidance: 1) identifying a search strategy; 2) determining inclusion and exclusion criteria; 3) screening papers; 4) assessing methodological quality; 5) extracting data; and, 6) synthesizing results [23].

### Search strategy

Two search approaches were used: 1) electronic database searching; and 2) grey literature searching. A three concept database systematic search strategy was developed by a librarian from Public Health Ontario’s (PHO) Library Services team, which included controlled vocabulary and natural language keywords related to the concepts of health promotion (e.g., “health promotion”, “public health”, “health education”), programs (e.g., “program”, “campaign”, “initiative”) and sustainability (e.g., “sustainable”, “routinization”, “long term implementation”). Searches were limited to articles published in English after 1998. The initial search strategy was developed using the MEDLINE database. The MEDLINE search strategy was peer-reviewed by other members of PHO’s Library Services team. The search syntax and controlled vocabulary terms in the MEDLINE search strategy were then translated for execution in secondary databases. The final search strategies were run in the following five databases on June 18, 2018: 1) Ovid MEDLINE; 2) Ovid PsycINFO; 3) EBSCOhost CINAHL plus with Full Text; 4) EBSCOhost SocINDEX with Full Text; and 5) Scopus.

A grey literature search was conducted on July 7, 2018. It included grey literature repositories, custom web search engines, and a general web search (see Additional file 1). Natural language keywords from the MEDLINE search were streamlined and adapted to develop search strings for identifying grey literature. Grey literature searches were run using the following search tools: New York Academy of Medicine Grey Literature Report; two custom search engines (one which searched the websites of Canadian provincial and regional health authorities and public health units; and one which searched the websites of international public health agencies and health authorities), and a web search engine (Google Canada). The grey literature strategy expanded the search beyond academic publishing, allowing for greater scope.

The database and grey literature searches were updated on June 26, 2019 in order to capture new papers that were released following our initial search. No changes to the search strategy were made when search results were updated. See Additional file 1 for full search strategy, including MeSH terms.

### Inclusion and exclusion criteria

Published and grey literature papers were included if they were published in English during the last 21 years; were about the sustainability of a HPP such as chronic disease health interventions in public health or a related setting (e.g., community-based, non-governmental or governmental organizations); explicitly used or referenced a sustainability framework or model to ground their HPP or guide their work; specifically reported on facilitators and/or barriers (or equivalent terms such as promote/inhibit) for the sustainability of a program; offered synthesis level research or primary level studies that included assessments of program sustainability and outcome data related to facilitators and barriers of program sustainability. The 21 year time frame was based on preliminary searches conducted to inform the strategy, and a decision made to coincide with the publication of Shediac-Rizkallah and Bone’s (1998) study, which continues to be cited today. For our review, we used Shediac-Rizkallah and Bone’s definition of sustainability, which defines it in three ways: 1) maintenance of health benefits from a program; 2) continuation of program activities within an organization; and 3) capacity building in the recipient community [11]. We focused our review on the second part of the definition, “continuation of program activities within an organizational structure”. Papers that addressed sustainability as it relates to parts 1 or 3 of the definition were excluded. We chose the second part of the definition because it focuses on the sustainability of program activities. A program was considered a HPP if it met the definition from the Ontario Public Health Standards, which states that HPP are public health programs designed to achieve program outcomes that improve “population health outcomes by reducing preventable disease, injury or death, and taking action on health inequities” [2].

Exclusion criteria included non-English language papers published before 1998, and those from countries outside of the Organisation for Economic Co-operation and Development (OECD). Papers were excluded if they were about sustainable development, sustainable food systems, or environmental sustainability; focused on sustainable organizations or sustaining systems or sectors (e.g., health care system, nursing); the program took place in a clinical or hospital setting; focused on sustainability of a partnership or coalition with no focus on program delivery; focused on sustainability of program impacts (e.g., continued health benefits for individuals after the initial program ends) and continued capacity of a community to develop and deliver HPP; no outcome data were provided; and commentaries and editorials.

The two authors (AB and SH) developed the inclusion and exclusion criteria at the outset of the project.

### Screening and selection of papers

#### Electronic data base

Titles and abstracts of the identified papers from the search were screened by two authors (AB and SH), who first independently screened 20% of search results for inclusion and had an agreement of 86.6%. The remaining 80% was split in half with each half screened independently by one of the two authors (AB and SH). For full text screening, two authors (AB and SH) first independently screened 20% of the articles and had agreement of 90%. The remaining 80% was split by 60% (AB) and 40% (SH) and screened independently. Any discrepancies were resolved by discussion until consensus was reached. For two articles, a third reviewer was consulted to apply the criteria and discuss ratings with both authors until consensus was reached.

#### Grey literature

Titles and abstracts of all grey literature search results were screened for inclusion independently by the two authors (AB and SH). All included papers were full-text screened by independently by both authors (AB and SH) and consensus was reached on all discrepancies through discussion.

### Quality assessment

#### Electronic data base

Methodological quality was assessed independently by a research coordinator (TO) who reviewed all included papers, and by two authors (AB and SH) who each reviewed half of the papers as a second reviewer, using appropriate criteria in regard to the design of the included papers. The Health Evidence Quality Assessment Tool for systematic reviews and meta-analyses was used for systematic reviews [24]. Papers that used qualitative methodology were appraised using the Critical Appraisal Skills Programme for qualitative methodology checklist [25]. A single cohort study was assessed using the Newcastle-Ottawa Scale for Cohort Studies [26]. All discrepancies on individual quality ratings were discussed until consensus was reached. The selected tools allowed the researchers to rate papers as strong (8–10), moderate (5–7) or weak (0–4) based on their methodological rigour, transparency and biases. All papers rated weak were excluded from this review, with the exception of one, due to its seminal contribution to the topic area and the fact that it remains highly cited across a 21 year time frame [11]. The ratings displayed are the final ratings agreed via consensus discussion by both authors (AB and SH) with the independent appraiser (TO) who critiqued all papers. Additional file 2 shows the ratings of the included studies listed as weak, moderate, and strong.

#### Grey literature

To assess the quality of the grey literature, a quality assessment tool was developed by the authors (AB and SH) using combined criteria from Caldwell [27] and Bergeron [28] for guidance. It included the following questions:

1. Is the methodology identified? If not, could the methodology be found within two internet clicks to additional resources?
2. Are the authors credible? (e.g., document is published by a university, government agency, author has other published work)
3. Is the rationale for the resource clearly identified? (e.g., a purpose statement or research question)
4. Is the methodology clear and transparent?
5. Did the authors use a sustainability framework? If they created their own, is it clear, and is it clear how the various components interact?
6. Are the results transferable? (e.g., to other sectors or settings)
7. Is it clear how the content could be used by practitioners?

Questions were answered with a scoring of ‘yes’ or ‘no’. If ‘no’ was answered for either of the first two questions, the paper was excluded. Remaining papers were screened with the remainder of the questions for a final score out of 6. Papers scored below 1–2 were considered weak, 3–4 moderate, and 5–6 strong. Moderate and strong papers were included and weak ones were excluded. Methodological quality was independently assessed by two authors (AB and SH) who each reviewed all papers. Any discrepancies were discussed until consensus was reached. Quality appraisal results for included grey literature are found in Additional file 2.

### Data extraction

A data extraction table was drafted to meet the research focus of this project, and refined by discussion between the authors. Two authors (AB and SH) tested and discussed the table, which created a guide for data extraction and instructions for completing each item to ensure consistency. Two authors (AB and SH) independently conducted data extraction for all included papers to ensure that all appropriate data were extracted from the included papers. Information extracted from each paper included: author and year of publication; study design; methods; purpose; objective or research aim; definition of sustainability; sustainability framework; barriers to sustainability; facilitators to sustainability; implications for the program planning process; conclusion; implications for practice; and future directions or research. Study limitations were also extracted and recorded as part of the conclusion column. Extraction tables from both authors were reviewed line by line and a harmonized data table was created (see Additional file 3). All discrepancies were discussed and consensus was reached. Authors of the included papers were not contacted to validate data.

### Data analysis

We conducted a deductive thematic analysis of our data, as per the definition provided by Vaismoradi [29]: “a method for identifying, analysing and reporting patterns (themes) within data.” A deductive thematic analysis involves the search for and identification of common threads, and comparing them across included studies [29]. After completion of the thematic analysis, we used Carroll’s [30] ‘best fit’ framework synthesis methodology to code the data. This method involves using an *a priori* framework and coding data from the review’s included papers against that thematic or conceptual framework [30]. Data that cannot be accommodated within the framework require interpretation using thematic analysis techniques, with new themes generated from data not captured by the *a priori* framework.

Schell’s [5] nine domain framework was used to code barriers and facilitators extracted from the included papers. This framework was the most recent and comprehensive framework identified within the health promotion sustainability literature. Barriers and facilitators that could not be coded within one of the nine domains were discussed by two authors (AB and SH) and additional codes were developed for them via consensus. The new codes allowed for the identification of new factors based on frequency. For this review, we define a factor as a collection of related barriers and facilitators. Frequency relates to the number of times the barrier/facilitator appears in the extraction results.

Once coding of all barriers and facilitators identified in the data extraction table was complete, they were examined for relevance and importance. Relevance was determined by analysing to what extent the factors were present in PHO’s 6-step approach for planning HPP [31], an evidence-based process used to inform and guide health promoters and public health practitioners. Step descriptions from PHO’s Program Planning Workbook were examined to determine commonalities between them and the identified factors. Factors that were similar were rated as more relevant. One author (AB) re-read all included articles and coded elements of each step with the factors.

Importance was coded by one author (SH) by re-reading all included articles and searching for instances where the authors of the included papers directly reported that a barrier or facilitator or multiple ones, were particularly important to the sustainability of the HPP. For example, authors stated that the barrier or facilitator was “the most important facilitator” [32], “integral to sustaining practice” [19], or “key to sustaining the HPP” [33]. Each of these were then coded as important.

All data were compiled into one table (See Additional file 4) and ranked into one of three tiers, set out according to frequency of mention, mentions of importance and mentions of relevance assessed for each barrier or facilitator.

## Results

**Fig. 1:**
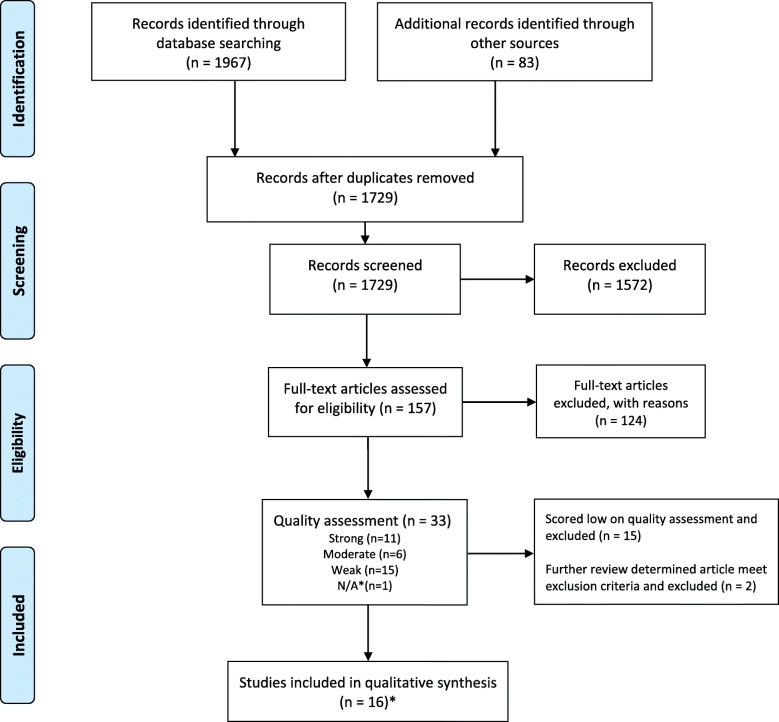
PRISMA flow chart diagram

The PRIMSA flow diagram reported in Fig. 1 details the process of identification and selection of papers for inclusion. Our search strategy identified 1729 papers in the peer reviewed and grey literature. Of these, 157 were selected for full text review. One hundred and twenty four (124) were excluded as they did not meet our inclusion criteria.

### Summary of quality assessment

A total of 33 papers were appraised for methodological quality, as described in the methods section. Seventeen published papers from electronic databases were assessed. Of these, six papers were rated as strong, five as moderate, and six as weak. The papers rated as weak either had methodology that rated as weak according to the quality assessment tool, or the methodology was not reported, and therefore could not be assessed. Following quality appraisal, a further two papers were re-assessed according to the inclusion criteria and removed.

Of the 14 grey literature papers assessed, nine were excluded as no methodology was included or could be found within two internet clicks. Four articles were rated as strong and one [20] moderate, as the components of their framework were not clearly defined and it was not clear if the results were transferable or how the framework could be used by others.

A total of seventeen papers scored low in quality and were excluded from the synthesis, with the exception of the paper by Shediac-Rizkallah and Bone [11], due to its importance to the field over time. After removal, 16 papers were included in the review.

### Overview of selected papers

Of the 16 included papers, there were six reviews (four literature reviews, one systematic review and one systematic narrative synthesis), seven primary studies, two workbooks, and one sustainability framework. The targeted outcomes of HPP studied were varied, and included diabetes [32], obesity [15], food insecurity [20], falls prevention [34], adolescent health [10, 35] and asthma [36]. Other included papers examined the sustainability of health education research programs in Aboriginal communities in Canada [18], the sustainability of evidence-based programs in disadvantaged communities [19], and sustaining a physical health promotion intervention in community mental health organisations [21]. The papers were from several countries, including the United States [5, 10, 16, 33, 36], Australia [17, 20, 34], Denmark [21], Canada [18], the United States and Canada [12], and multiple countries [15, 19]. One article did not specify their search terms and so country of origin/inclusion could not be identified [11]. Table 1 summarizes characteristics of included papers. Further details can be seen in Additional file 2.

**Table 1:** Summary of included papers

Study	Definition of Sustainability	Sustainability Framework used/developed	Factors
Altarum Institute 2009 [16]	Shediac-Rizkallah and Bone, 1998 [11]	Conceptual Model for Evaluating the Sustainability of Community Health Initiatives (Beery et al. 2005 [37])	• Communications • Funding (3) • Organizational capacity (2) • Partnerships • Program evaluation (3) • Strategic planning (3) • Champion • Political support • Fit/alignment
Carstensen et al. 2019 [21]	Scheirer 2013 [38]	Normalization Process Theory (NPT) May and Finch 2009 [39]	• Organizational capacity (6) • Program adaptation • Program evaluation • Fit/alignment (3) • Capacity building • Policy • Socio-economic/political factors • Mandatory municipal action plans for users (clients)^a^
Casey et al. 2009 [17]	Shediac-Rizkallah and Bone, 1998 [11]	Assessing if a program is likely to be sustained checklist from “Indicators to help with Capacity Bulling in Health Promotion” [40]	• Partnerships (2) • Capacity building (2) • Champion • Fit/alignment (3) • Funding (3)
Elsworth and Astbury 2005 [20]	A sustainable program is one that has become routinized in an organization as well as standardized within policy making institutions.	Multi-Level Model of Project Sustainability	• Strategic planning • Champion • Organizational capacity • Affordance: the opportunity provided by the policy or program for local innovation^a^ • Program access^a^
Garst et al. 2017 [32]	Scheirer and Dearing, 2011 [41]	Framework for Sustainability of Translational Research Projects (adapted from Scheirer 2011 [41])	• Communications (3) • Partnerships (5) • Strategic planning (2) • Organizational capacity (3) • Program evaluation (2) • Socio-economic/political
Hill et al. 2011 [34]	Sustainability refers to the long term continuation of effective programs, or, where there is a set of activities aimed at achieving the programs objectives that are incorporated into the organizations routines.	12 factors for sustainability and sustainability checklist	• Communications • Organizational capacity (5) • Partnerships (2) • Public health impacts • Program adaptation • Strategic planning (1) • Capacity building • Champion (2) • Fit/alignment (2) • Program implementation • Funding (2)
Hodge and Turner 2016 [19]	Sustained program implementation is whether a program operated over multiple years.	Sustained Implementation Support Framework for Evidence-Based-Programs.	• Funding(3) • Organizational capacity (17) • Partnership (7) • Public health impacts • Political support (3) • Program adaptation • Program evaluation (3) • Strategic planning (3) • Capacity building (4) • Champion • Fit/alignment (2) • Program implementation (5)
Office of Adolescent Health 2014 [35]	Effectively leveraging partnerships and resources to continue programs, services and or strategic activities that result in improvements in the health and wellbeing of adolescents. OAH recommends that grantees create their own definition of sustainability.	Framework for Program Sustainability	• Funding (2) • Partnerships (2) • Political support • Strategic planning (2)
Office of Adolescent Health 2017 [10]			• Communications • Funding (2) • Organizational capacity (3) • Partnerships (3) • Program adaptation • Strategic planning (3) • Champion • Capacity building • Fit/alignment
Paine-Andrews et al. 2000 [33]	The extent to which community changes facilitated by the initiatives remained in place after grant termination and the extent to which the initiatives themselves remained in place after grant termination.	Model for Institutionalizing Health Promotion Programs (Steckler and Goodman 1989 [42])	• Organizational capacity (3) • Partnerships (2) • Public health impacts (2) • Strategic planning • Champion
Sadof et al. 2006 [36]	Sustainability is defined as the continuation of the central elements of the Inner City Asthma Intervention program.	Framework for Conceptualizing Program Sustainability (Shediac-Rizkallah and Bone, 1998 [11])	• Communications (2) • Funding • Organizational capacity (2) • Partnerships (5) • Political support (2) • Program evaluation (7) • Champion
Scheirer 2005 [12]	Shediac-Rizkallah and Bone, 1998 [11]	Framework for Conceptualizing Program Sustainability (Shediac-Rizkallah and Bone, 1998 [11])	• Funding (2) • Organizational capacity (5) • Partnerships (4) • Public health impacts (2) • Program adaptation (2) • Program evaluation (1) • Strategic planning • Capacity building • Champion • Fit/alignment (5) • Program implementation (1) • Funder priorities^a^
Schell et al. 2013 [5]	The ability to maintain programming and its benefits over time. Sustainability capacity is defined as the existence of structures and process that allow a program to leverage resources to effectively implement and maintain evidence based policies and activities.	Framework of public health program capacity for sustainability	• Funding stability • Political support • Partnerships • Organizational capacity • Program adaptation • Program evaluation • Communications • Public health impacts • Strategic planning
Shediac-Rizkallah and Bone 1998 [11]	Sustainability is a global term used to refer to the general phenomenon of program continuation. Three perspectives on sustainability: 1) maintain health benefits achieved through the initial program 2) continuation of the program activities within an organization structure and 3) building the capacity of the recipient community	Framework for Conceptualizing Program Sustainability	• Funding (2) • Organizational capacity • Partnerships (2) • Program evaluation • Strategic planning (5) • Capacity building • Champion • Socio-economic/political factors (3) • Funder priorities (2)^a^
Whelan et al. 2018 [15]	Sustainability of obesity prevention is defined as changes in behavioural determinants and/or BMI at least 12 months post the initial impact has been demonstrated.	Ten Key Elements of sustainability [43]	• Communications • Funding • Organizational capacity (6) • Partnership (4) • Program adaption • Program evaluation (2) • Capacity building (2) • Champion • Fit/alignment (1) • Policy^a^
Wisener et al. 2017 [18]	A sustainable program is: 1) receptive to change and adaptable 2) an innovative strategy that provides continued benefit 3) fully integrated into normal operations post-project funding 4) of benefit to diverse stakeholders	Seven factors that promote or inhibit the sustainability of Community Learning Centres	• Funding (2) • Partnerships (4) • Capacity building (3) • Champion (2) • Fit/alignment (3) • Program access (2)^a^

^a^Tier 3

#### Definitions of sustainability

All 16 included papers provided a definition of sustainability. Eleven papers developed their own definitions [5, 10, 11, 15, 18–20, 33–36]. Four papers [12, 16, 17, 36] used Shediac-Rizkallah and Bone’s (1998) definition of sustainability, while two other papers [21, 32] used definitions by Scheirer [38, 41]. Table 1 provides an overview of each paper, including the definition used, the framework or model used and the factors coded from it.

While each paper included in this review cited a definition of sustainability to guide their own work, many referred to inconsistencies in how HPP define sustainability or that in many cases, HPP did not define sustainability at all [11, 15–18, 21].. The most consistent definition of sustainability from the included papers is program continuation beyond financial security [10, 15, 19, 20, 35]. Shediac-Rizkallah and Bone [11] cite three perspectives on what specifically can be sustained:

1. Individual level: maintaining health benefits for individuals after initial program funding ends, particularly continuing to achieve beneficial outcomes for new clients.
2. Organizational level: continuing program activities within an organizational structure and ensuring that program goals, objectives, and approaches adapt to changing needs over time.
3. Community level: building the capacity of the community to develop and deliver program activities, particularly when the program found success through a community coalition or community capacity-developing process.

#### Sustainability frameworks

All of the 16 included papers cited a sustainability framework. Nine papers used existing frameworks [15–18, 21, 32, 33, 36, 41] while seven developed their own [5, 10, 11, 19, 20, 34, 35]. Two grey literature papers from the Office of Adolescent Health [10, 35] cited the same framework. Sadof (2006) and Scheirer (2005) used Shediac-Rizkallah and Bone’s 1998 framework. In total, 13 unique frameworks were cited. See Table 1 for details on the included studies and the framework used in each one.

There were two types of frameworks cited in the papers: most depicted sustainability in terms of barriers and facilitators for sustainability [5, 10, 11, 15, 17–21, 32] while two conceptualized sustainability as a step-by-step process. The framework by Beery et al. [42] cited in Altarum [16], and the framework by Steckler and Goodman [37] cited in Paine-Andrews [33] are the only frameworks that suggest a step by step process for transitioning programs towards sustainability. Only one of the included papers, the *Community falls prevention program sustainability guidelines and workbook* [34], provides guidance for planning HPP sustainability at the outset.

One of the included papers, Schell et al. [5], presented the framework which we subsequently used for initial coding of the barriers and facilitators extracted for this study. Following its 2013 publication, it formed the basis of the Program Sustainability Assessment Tool, which has been validated and assessed for reliability [14] and is commonly used in the public health field today.

#### Barriers and facilitators

For the purpose of this paper, a *barrier* inhibits or prevents the sustainability of a HPP while a *facilitator* promotes sustainability or is associated with sustainability. In total, 83 barriers and 191 facilitators related to sustainability of HPP were extracted from the included papers. Since the Schell et al. framework was used to code barriers and facilitators, the barriers and facilitators from their framework were not included in the analysis results. Two papers from the Office of Adolescent Health [10, 35] cited the same facilitators which were only counted once. Wisener et al. [18] cited seven considerations which promote or inhibit sustainability and were therefore counted as both barriers and facilitators in the results.

#### Factors for HPP sustainability

Twenty-three barriers and 54 facilitators could not be coded into the domains of the Schell framework. As such, five additional factors were created by the authors to summarise and describe them. This results in a total of 14 factors for sustainability being identified: nine from the Schell framework; and five new ones based on the results of our analysis. Five barriers and five facilitators could not be coded into either the Schell domains or the five new factors, and were considered outliers.

Factors were grouped into one of three tiers based on relative prominence in the past 21 years of health promotion sustainability literature. Tier 1 had codes with a frequency of ≥15 up to 54 and were heavily coded as important and/or relevant. These factors were considered to be the most influential for program sustainability (see Additional file 4). Tier 2 had factors with a frequency of ≥5 to ≤10, with several being coded specifically for importance and/or relevance. These were considered relevant factors to program sustainability. Tier 3 included barriers and facilitators with a frequency of ≤4, three of which each had one mention of importance, and no mentions of relevance and were determined to not be factors for program sustainability. Based on the above analysis, factors for HPP sustainability were identified. Table 2 outlines the factors for sustainability, grouped by tier and in order of most important to least.

**Table 2:** 14 factors for health promotion program sustainability

Tier 1	Organizational capacity
	Partnerships
	Strategic Planning
	Funding
	Fit/alignment^a^
	Program Evaluation
	Capacity Building^a^
	Champion^a^
Tier 2	Communications
	Program Implementation^a^
	Political Support
	Program Adaptation
	Public Health Impacts
	Socio-economic/political factors^a^
Tier 3	Program access factors
	Funder priorities
	Policy
	Affordance
	Tailored activity plans for individual clients

^a^New factors identified in this review

The following sections discuss the most critical factors for sustainability from our analysis; tier 1 and 2 factors. The most important barriers and facilitators as they relate to the main factor are discussed in each section. Given the breadth of the results, it is not possible to discuss all 14 factors in detail. Please see Additional file 4 for more information on analysis results.

### Organizational capacity

Organizational capacity refers to “having the resources needed to effectively manage the program and its activities” [5]. This was the most highly coded factor in terms of frequency and importance [10–12, 15, 16, 19–21, 32–34, 36]. Staffing issues were a major barrier to sustainability, namely a lack of qualified staff, [15, 19, 34] difficulties in recruiting and retaining staff, [36] and high staff turnover [12, 15, 19, 32]. Leadership and management support was a key facilitator as well as a barrier: strong leadership and buy-in from senior management was a facilitator while lack thereof acted as a barrier to sustainability [10, 19, 21, 34]. Identifying, engaging and developing internal leaders, [10] building an internal leadership team rather than having one individual lead, [35] and having senior leaders articulate organizational values and vision through action, all improved HPP sustainability [19].

### Partnerships

Schell describes partnerships as the “connection between program and community” [5]. This factor was mentioned in all of the 16 included papers in this review, indicating the strong relationship between partnerships and program sustainability [10–12, 15–21, 32–36]. The included papers described partnerships with a wide variety of stakeholders from a variety of sectors (such as health and government), communities, and staff from the HPP [10, 18, 19, 44]. A network of organizations can help to advocate for sustainability [17], as well as contribute to sustainability by providing additional resources [34], bringing specific skills and knowledge to the partnership [34], and supporting service delivery [10]. Programs cannot be sustained if the people responsible for running, supporting or using the program do not see its value [34]. Collaborating with community, mobilizing community, and using participatory approaches also contribute to sustainability [11, 15]. The level of involvement of partners and communities in the HPP is important: close involvement [32], participatory planning [19], and a sense of ownership increased community capacity to sustain the program [11] whereas lack of partnership, engagement, local buy-in and uptake of the program can inhibit sustainability [18, 19, 35].

### Strategic planning

Strategic planning was frequently mentioned as an important factor for program sustainability [10–12, 16, 19, 20, 32–35]. It refers to “the process of defining program direction, goals and strategies” [5]. Purposefully considering if the program could be integrated into existing organizational structures, routines and roles improved the likelihood of sustainability as existing structures and processes make integration of a new program easier [34]. Early planning for sustainability [10, 12, 19, 34, 35] for instance allocating funds for it early on, and planning to regularly assess it at key periods during the HPP lifecycle improved sustainability.

### Funding stability

Funding plays an important role in program sustainability [10–12, 15–19, 34–36]. Schell defines this factor as “making long-term plans based on a stable funding environment.” The importance of securing funding from multiple and diverse sources was frequently mentioned [10, 12, 16, 36]. Lack of stable funding impeded sustainability: in some cases it was impossible to continue programming without funds [17, 34]. Casey et al. [17] also cited that the search for funding could distract program staff and the program could suffer as a result, and staff may have limited capacity to devote to sustainability as they are concentrating on implementing the program.

### Fit/alignment

Fit/alignment was developed by the authors of this paper as an important factor based on numerous mentions of its frequency and importance in the included papers [10–12, 15–19, 34–36]. This factor refers to alignment between the HPP and the organization’s mandate and core business [12, 19, 34]. It also can refer to alignment between the HPP and community need [34], priorities [18] and community opinion [17]. Programs that could readily fit into existing tasks/procedures and contribute to the organization were more likely to be supported, and therefore sustained [41].

### Program evaluation

Program evaluation was identified as another key factor for sustainability [11, 12, 15, 16, 19, 21, 32, 36]. It refers to the monitoring and evaluating of process and outcome data associated with program activities [5]. In particular, data collection and analysis, namely having the appropriate resources to collect sound data and analyse it, was found to be important for sustainability [36]. A possible reason for this is that it allows for determining whether the HPP is being carried out effectively, and if it is worth sustaining [11]. As well, lack of proper evaluation methods meant not being able to report clearly on project findings and results. This impacted sustainability because it made it difficult to demonstrate the value of the HPP to stakeholders and key individuals who have the ability to make decisions regarding further funding and integration into operational plans [32]. Also, the degree to which the HPP could be evaluated for its implementation and effectiveness outcomes, and whether the findings were reported back to program staff and used to align program delivery in the field impacted sustainability, because adapting a program to objective data ensured it was relevant and met the needs of its recipients [19].

### Capacity building

We identified capacity building as a new factor for sustainability [10–12, 15, 17–19, 21, 32, 34]. It refers to creating conditions for success at individual, program, agency or system levels and involves development of sustainable skills, organizational structures and commitment with a focus on prolonging the HPP [45, 46]. In particular, directed effort toward ongoing staff training and workforce development, such as skill development related to the HPP, improved sustainability [15, 32].

### Program champion

Nearly all of the included papers highlighted the use of a program champion [10–12, 15–18, 20, 33, 34, 36], therefore we identified it as a new factor. A program champion is an influential individual who acts as an advocate for the HPP [11]. They often enthusiastically advocate for the needs of the program, particularly to help secure resources for its continuation [12, 18]. In many cases they are powerful people in leadership positions as well as key partners or role models [15]. Using a champion for stakeholder engagement was beneficial for sustainability. Specifically, a credible, enthusiastic individual from within the organization who can engage management and key staff for financial support and advocate for organizational policies that support the HPP [34]. A champion at the executive level was found to be particularly useful for enhancing sustainability because they were able to advocate for the programs needs and secure resources for its continuation [12]. In one case, program failure was attributed to lack of a champion [34].

#### Additional factors

Tier 2 factors did not score as high for frequency, relevancy and importance in our analysis, however they were associated with program sustainability.

### Communications

This factor played an important role in HPP sustainability [10, 15, 16, 19, 32, 34, 36]. It refers to strategic dissemination of program outcomes, results and activities with stakeholders, decision makers and the public [5]. In particular, regular communication with stakeholders, policy makers and partner organizations improved sustainability because it allowed for sharing of experiences and problem solving during program implementation, while helping to determine which factors specific to the community were needed for sustainability [32].

### Program implementation

The amount of attention given to program implementation played a role in program sustainability [12, 19, 34]. It was identified as a new factor. In particular, when external partners were not involved in the host agency’s effort to implement the program, sustainability suffered [12, 19]. Overall, the greater the reach of the program during the implementation phase, combined with the use of practitioner experience during this phase, improved HPP sustainability [19, 34].

### Political support

Political support should be given specific consideration when planning a HPP [16, 19, 35, 36]. It refers to the internal and external political environment which influences program funding, initiatives, and acceptance [5]. Particular attention given to assessing and understanding the local political climate in which the HPP is being implemented is likely to impact its success. More so, garnering local politician support will improve likelihood for sustainability because having someone who can promote and advocate for policies that support the HPP can improve sustainability outcomes [16, 19, 36].

### Program adaptation

This was another highly cited factor [10, 12, 15, 19, 21, 34]. It refers to the program’s ability to adapt and improve in order to ensure effectiveness [5]. The ability to easily adjust the HPP to local operating conditions and the broader environment in regard to organization changes and new research knowledge while still maintaining critical program components improved sustainability [19, 34]. Giving specific attention to creating a program that is modifiable over time was also important [12].

### Public health impacts

Clearly demonstrating public health impacts of the HPP improved sustainability [12, 19, 33, 34]. In particular, when the benefits of the HPP were clear to program recipients, and were actively promoted, sustainability was enhanced [12, 19]. This was also thought to influence the way the program was perceived by the practitioner, such that if they had a clear understanding of its benefits, they did a better job of implementing it [19].

### Socio-economic and political climate

This was a new factor for sustainability identified by this review [11, 21, 32]. It is not to be confused with ‘political support’ which relates directly to advocacy to and support from politicians. It captures elements that may be outside of the control of practitioners but nevertheless would have an impact on sustainability, and should be considered during planning and throughout the HPP lifecycle. For example, changes in government, short budget cycles, internal political pressures, and the time it takes to develop a policy [11, 32]. As well, competing issues such as poverty, unemployment and crime could prevent a HPP from being sustained [11]. Also, a less favourable environment for sustainability, such as deteriorating economic conditions and weakening government institutions (e.g., high- versus low-developed countries) is another important consideration [11].

#### Tier 3

These barriers and facilitators scored low for frequency, importance and relevance and were determined to not be factors for program sustainability in our review. However, they should not be overlooked altogether as they may play a role in sustainability depending on the specific HPP and local context. They included: considerations for program access [18, 20]; funder priorities [11, 12]; policy [15, 21]; affordance, described as a “dynamic relationship or ‘transaction’ between an individual and a location in the environment” [20]; and tailored activity plans for individual users [21].

## Discussion

This review provides health promotion and public health practitioners with an updated and practical inventory of factors that impact HPP sustainability based on a comprehensive and systematic review of published and grey literature. The results of this review support the findings of key papers in the health promotion program sustainability literature in regard to the factors for sustainability [5, 11, 12], while adding new information to the body of knowledge on this topic. The literature search for this review covered the last 21 years, allowing for a comprehensive assessment of the sustainability literature relevant to health promotion. More than half of the included papers were rated as ‘strong’ methodological quality with the remainder being ‘moderate,’ suggesting that the body of literature on this topic is comprehensive and methodologically sound, in addition to being relevant and applicable to the current context.

We identified 14 key factors for the sustainability of HPP that could be embedded into the program planning process. Nine of these factors were from Schell’s framework [5], and five were identified as new factors. Although we have reported on factors for sustainability as separate and distinct from one another, it should be noted that they do not always exist in isolation, and can be complex and interrelated. In this section, we discuss the factors as they related to one another, rather than in order of frequency.

Organizational capacity was the most highly coded factor in terms of frequency and importance, meaning that this factor heavily influences program sustainability. Organizations must ensure that the capacity required to fully implement and sustain their HPP is available prior to implementation of the program. Staffing, leadership and management support could be considered during the program planning stage. Factors related to organizational capacity include program implementation, as it lays the foundation for sustainability. Additionally, the factor of funding stability must be prioritized. Dedicating resources and staffing to this is one way to achieve this [10, 15]. Having multiple strategies to obtain funds [12] is another.

Given the strong relationship between partnerships and sustainability, partnerships with communities, organizations and stakeholders from multiple sectors should be embedded into program planning. Strategies for effective partnerships include engaging with partners who have a shared vision [10] and similar missions [32], dividing tasks between partners [32], using participatory approaches [15, 19], and building sustainability into partnership agreements [10]. This factor had the highest mentions in relevance, reinforcing its importance in program planning. A specific strategy to involve partners is to engage them as program champions. Practitioners should give particular consideration to strategically and purposefully identify and secure the support of a champion during the planning stages of a HPP, prior to program implementation.

Strategic planning was an important factor for program sustainability and considerable time and attention should be given to it. Planning the HPP and its associated activities around an established theory or framework, for example the trans-theoretical model, helps structure the HPP activities around validated approaches [31], thus contributing to sustainability. Assessing the environment and local context in which the program will be implemented prior to it beginning is an important component of the planning process [10, 31]. Practically, this can be done by conducting a situational assessment or a Strengths, Weakness, Opportunities and Threats (SWOT) analysis. Practitioners should give consideration to socio-economic and political climate when conducting a situational assessment as it will likely impact the success of their HPP. As part of the situational assessment, consider the fit/alignment of the program. Lack of fit/alignment with the organization, partners and community is as likely to inhibit sustainability as fit/alignment would promote sustainability. In addition, potential adaptability of the program should be considered. Practitioners should ensure that their HPP is modifiable to meet the evolving needs of their local context, community, and health system.

Program evaluation contributes to program sustainability in several ways. Identifying and selecting specific outcomes and indicators to measure sustainability is important for overall sustainment, as this clarifies for practitioners what needs to be sustained, how elements of the HPP can be sustained, and indicates when sustainability has been achieved. As demonstrating the public health impacts of the HPP was also identified as a factor for sustainability, particular focus should be given to data collection and analysis, and routinely planning for evaluations during the program lifecycle. Having the appropriate resources to ensure sound data collection and analysis is important because it allows for objectively demonstrating to stakeholders and funders whether the program is being implemented as intended, is having its intended impact, and if it is worth sustaining. As such, practitioners should give ample attention to how they will evaluate the sustainability of their HPP, as well as to securing the appropriate tools to collect and analyse data. It may be necessary to prioritize data collection, if there is limited time or resources.

Another key consideration for sustainability is the role of capacity building services for program staff. Capacity building is an important factor because it provides program staff with the necessary skills and knowledge to properly implement the HPP. Attention should be given to providing staff with ongoing technical assistance to support them as they implement the HPP. As well, providing educational opportunities that further their understanding of the issue being addressed by the HPP improves buy-in and quality of service delivery. Providing ongoing skills and professional development opportunities improves staff retention which allows for program continuity and improved organizational memory. When implemented strategically capacity building can help during periods of uncertainty by providing the necessary support system for staff and participants. It also helps improve staff retention, which is valuable for sustainability because it improves organizational memory.

This review provides several practice recommendations in addition to the 14 factors. Particular attention should be given to identifying which specific component of the HPP should be sustained. Shediac-Rizkallah and Bone’s definition of sustainability suggests that three components could be sustained: health impacts of the program; the program itself; or community capacity to sustain the program. Second, the sustainability literature commonly cites that there is variation in how sustainability is defined, when it is defined at all. This creates confusion and suggests a lack of conceptual understanding of what sustainability is and what is required to sustain programs. Adding to this, a number of terms related to sustainability are used in the literature [7]. For example, ‘institutionalization’ refers to the continuation of a program or program activities within an organization beyond the funding period [37], while ‘routinization’ refers to the program (or its components) becoming established on a durable basis (i.e., routine) [7]. This issue has plagued the health promotion field for decades as the lack of a clear definition makes it difficult to plan, implement, and evaluate whether sustainability has been achieved. Therefore, it is recommended that practitioners define sustainability as it relates to their HPP prior to attempting to sustain the program. Practitioners could use or adapt a definition commonly-available in the literature, such as Shediac-Rizkallah and Bone [11] and Scheirer [12], or develop a definition that is specific to the program. The critical point is that sustainability needs to be clearly defined in some way. Once a definition of sustainability has been selected or developed, a sustainability framework or model can provide direction on how sustainability can be achieved and evaluated. This review identified 13 sustainability frameworks. Two grey literature workbooks [34, 35] provide step by step models and practical worksheets to assist practitioners in planning sustainable programs. Program planners should, within their local context, consider factors that are key to implementation and likely influence outcomes [47], and select a framework that best fits to use or adapt.

Further research could include additional work to validate the 14 factors identified in this review to assess which factors are most important and their applicability in different practice contexts. It may be useful to consolidate objective indicators for sustainability to help practitioners assess whether their sustainability efforts have been achieved. Also, given the number of frameworks and models that exist in the field, a concerted effort to organize health promotion and public health practitioners around a single set of measures to assess sustainability would be beneficial.

### Review limitations

There may be other relevant documents beyond the published articles and grey literature searches, not available in the public domain or published in other languages on this topic, which could not be included in this review due to time and resource restrictions. Given this review’s focus on health promotion and public health, there is the possibility that our search strategy missed literature from related fields such as implementation science. However given the focus on health promotion and public health, this review assessed the state of literature closest to that context based on the most relevant and current papers. A potential limitation is the manner in which frequency, importance and relevance of facilitators and barriers was assessed. Although it served the purpose of our review, the assessment of relevance involved a subjective interpretation of the literature in some cases. However, including importance and relevance added to the depth and strength of our findings by allowing us to go beyond simply reporting results based on frequency of codes.

## Conclusions

This systematic review identifies 14 factors that influence the sustainability of HPP. Nine factors were previously identified in the sustainability literature: organizational capacity; partnerships; strategic planning; funding stability; program evaluation; communications; political support; program adaptation; public health impacts. Five new factors were identified: fit/alignment; capacity building; program implementation; program champions; and socio-economic and political climate. Specific barriers and facilitators as they relate to each factor were highlighted. This paper expands on established and persistent sustainability factors, adding new dimensions to for a more complete picture of program sustainability. A unique aspect to these findings is that they build on the existing literature by providing new considerations for the factors that impact HPP sustainability which can be used by health promotion and public health practitioners to promote the sustainability of their HPP. A strength of this review is the inclusion of grey literature, and the development of quality assessment criteria in order to provide transparency around the quality of the included papers.

The findings also highlight the need for practitioners to clearly define sustainability and identify what needs to be sustained early on during the planning process: the health benefits of the program, program itself (or its components), or capacity of the community to continue the program. In addition, practitioners should use a sustainability framework to guide their sustainability planning efforts; several were identified in this review. Given the importance of HPP in improving population health, reducing health inequities, and reducing health care costs, health promoters and public health practitioners should strive to prioritize the sustainability of their programs by actively planning for sustainability throughout program implementation and the program lifecycle. The factors identified in this systematic review could be incorporated into the design of HPP to increase their likelihood of sustainment.

## Supplementary information

**Additional file 1: Appendix A and B.** Search strategy examples. Full MEDLINE search strategy, and repository and general web search for grey literature.

**Additional file 2: Appendix C.** Results of quality assessment of the included 16 papers with final ratings.

**Additional file 3.** Characteristics of included papers.

**Additional file 4.** Weighting of factors for sustainability by frequency, importance and relevance.

## Data Availability

All data gathered by this review are included in the published article and its Additional Files. The secondary data (included papers themselves) which were analysed in this review is accessible through existing journals. All papers not available by open access were accessed via Public Health Ontario subscriptions. Subscriptions access restricts sharing all included papers via direct links however the papers are listed in References for access by readers.

## Abbreviations

HPP: Health Promotion Programs
OECD: Organisation for Economic Co-operation and Development
PHO: Public Health Ontario
SWOT: Strengths, Weaknesses, Opportunities, Threats
