# Supplementary material for: Sustainable by design: a systematic review of factors for health promotion program sustainability
Source: BMC Public Health. 2020 Jun 19;20:964. doi: 10.1186/s12889-020-09091-9 (PMC7304137; doi:10.1186/s12889-020-09091-9)
Supplement: Supplementary file 1 — Additional file 1: Appendix A and B. Search strategy examples. Full MEDLINE search strategy, and repository and general web search for grey literature. [file 12889_2020_9091_MOESM1_ESM.docx]

**Appendix A**

Ovid MEDLINE(R) Epub Ahead of Print, In-Process and Other Non-Indexed Citations, Ovid MEDLINE(R) Daily, Ovid MEDLINE and Versions(R) <1946 to June 13, 2018>

| # | Searches | Results |
| --- | --- | --- |
| 1 | Community Health Services/ or Health Education/ or Health Policy/ or Health Promotion/ or Preventive Health Services/ or Primary Prevention/ or Public Health Administration/ or Public Health Nursing/ or Public Health Practice/ or Public Health/ or United States Public Health Service/ or ("health authorit*" or "health department*" or "health education" or "health policies" or "health policy" or "health program*" or "health promot*" or "health unit*" or "population health" or "public health" or (communit* adj3 health*) or (disease* adj3 prevent*) or prevention or preventive).kf,kw,ti. or ("health authorit*" or "health department*" or "health education" or "health policies" or "health policy" or "health program*" or "health promot*" or "health unit*" or "population health" or "public health" or (communit* adj3 health*) or (disease* adj3 prevent*) or prevention or preventive).ab. /freq=2 | 663488 |
| 2 | Community Health Planning/ or Government Programs/ or Health Plan Implementation/ or Health Planning/ or Health Planning Guidelines/ or Healthy People Programs/ or Immunization Programs/ or Mandatory Programs/ or National Health Programs/ or Needle-Exchange Programs/ or Organizational Case Studies/ or Pilot Projects/ or Program Development/ or Program Evaluation/ or Regional Health Planning/ or Regional Medical Programs/ or State Medicine/ or Voluntary Programs/ or Weight Reduction Programs/ or Health Care Coalitions/ or ("action plan*" or "community challenge*" or "community partnership*" or "health plan*" or "sustainable health promotion action" or "tak* action" or "task force*" or (health adj3 action*) or approach or approaches or campaign* or coalition* or implement* or initiative* or innovation* or pilot* or policies or policy or program* or project*).kf,kw,ti. or (program or programs or programme or programmes).ab. /freq=2 | 1069332 |
| 3 | (sustainab* or sustainment or routinis* or routiniz* or "long term implementation" or "long term effectiveness" or "long lasting" or "voltage drop*" or "program drift*" or ((maintain* or continue* or continuing or continuation or last* or durab* or persist* or sustain*) adj5 (change* or benefit* or effective* or outcome* or behav* or fund* or program* or service* or resources or "long term" or "over time" or "extended period of time")) or endgame).kf,kw,ti. | 23804 |
| 4 | sustain*.ti. and sustain*.ab. /freq=2 | 11020 |
| 5 | 1 and 2 and (3 or 4) | 1142 |
| 6 | limit 5 to english | 1084 |
| 7 | limit 6 to yr="1998 -Current" | 963 |
| 8 | (exp Africa/ or exp Caribbean Region/ or exp Central America/ or exp Latin America/ or exp South America/ or exp Asia/ or Mexico/ or Developing Countries/) not (North America/ or exp Canada/ or exp United States/ or exp Australia/ or New Zealand/ or exp Europe/ or exp Developed Countries/) | 1030616 |
| 9 | 7 not 8 | 751 |
| 10 | "sustainable development goal*".ti,ab,kw,kf. | 1110 |
| 11 | 9 not 10 | 710 |
| 12 | *Environmental Health/ or exp *Climate Change/ or *Environmental Pollution/ or *Ecosystem/ or *Conservation of Natural Resources/ or *"Environmental Restoration and Remediation"/ | 79637 |
| 13 | 11 not 12 | 658 |
| 14 | remove duplicates from 13 | 649 |

PsycINFO <1806 to June Week 2 2018>

| # | Searches | Results |
| --- | --- | --- |
| 1 | Behavioral Medicine/ or Community Health/ or Health Behavior/ or Health Care Policy/ or Health Education/ or Health Promotion/ or Prevention/ or Preventive Medicine/ or Public Health Services/ or Public Health/ or ("health authorit*" or "health department*" or "health education" or "health policies" or "health policy" or "health program*" or "health promot*" or "health unit*" or "population health" or "public health" or (communit* adj3 health*) or (disease* adj3 prevent*) or prevention or preventive).ab,id,ti. | 248335 |
| 2 | After School Programs/ or Coalition Formation/ or Community Services/ or Educational Program Planning/ or Educational Programs/ or Government Programs/ or Home Visiting Programs/ or Mental Health Programs/ or Needle Exchange Programs/ or Outreach Programs/ or Program Development/ or Program Evaluation/ or School Based Intervention/ or Social Programs/ or ("action plan*" or "community challenge*" or "community partnership*" or "health plan*" or "sustainable health promotion action" or "tak* action" or "task force*" or (health adj3 action*) or approach or approaches or campaign* or coalition* or implement* or initiative* or innovation* or pilot* or policies or policy or program* or project*).id,ti. or (program or programs or programme or programmes).ab,id,ti. | 578498 |
| 3 | Sustainable Development/ or (sustainab* or sustainment or routinis* or routiniz* or "long term implementation" or "long term effectiveness" or "long lasting" or "voltage drop*" or "program drift*" or ((maintain* or continue* or continuing or continuation or last* or durab* or persist* or sustain*) adj5 (change* or benefit* or effective* or outcome* or behav* or fund* or program* or service* or resources or "long term" or "over time" or "extended period of time")) or endgame).id,ti. | 11227 |
| 4 | sustain*.ti. and sustain*.ab. /freq=2 | 4533 |
| 5 | 1 and 2 and (3 or 4) | 604 |
| 6 | limit 5 to english language | 584 |
| 7 | limit 6 to yr="1998 -Current" | 531 |
| 8 | (("Afghanistan" or "Africa" or "Algeria" or "Angola" or "Antigua" or "Argentina" or "Armenia" or "Asia" or "Azerbaijan" or "Bahamas" or "Bahrain" or "Bangladesh" or "Barbados" or "Barbuda" or "Belize" or "Benin" or "Bhutan" or "Bolivia" or "Botswana" or "Brazil" or "Brunei" or "Burkina Faso" or "Burma" or "Burundi" or "Cambodia" or "Cameroon" or "Cape Verde" or "Central African Republic" or "Central America" or "Chad" or "Chile" or "China" or "Colombia" or "Comoros" or "Costa Rica" or "Côte d'Ivoire" or "Cuba" or "Curacao" or "Cyprus" or "Democratic Republic of the Congo" or "Djibouti" or "Dominica" or "Dominican Republic" or "East Timor" or "Ecuador" or "Egypt" or "El Salvador" or "Equatorial Guinea" or "Eritrea" or "Ethiopia" or "Fiji " or "Flores" or "French Guiana" or "Gabon" or "Georgia" or "Ghana" or "Grenada" or "Guatemala" or "Guinea" or "Guinea-Bissau" or "Guyana" or "Haiti" or "Honduras" or "India" or "Indonesia" or "Iran" or "Iraq" or "Israel" or "Ivory Coast" or "Jamaica" or "Japan" or "Jerusalem" or "Jordan" or "Kazakhstan" or "Kenya" or "Kiribati " or "Kuwait" or "Kyrgyzstan" or "Laos" or "Lebanon" or "Lesotho" or "Liberia" or "Libya" or "Lombok" or "Madagascar" or "Malawi" or "Malaysia" or "Maldives" or "Mali" or "Marshall Islands " or "Mauritania" or "Mauritius" or "Melanesia" or "Mexico" or "Micronesia " or "Mongolia" or "Morocco" or "Mozambique" or "Myanmar " or "Namibia" or "Nauru " or "Nepal" or "New Caledonia" or "New Guinea" or "New Zealand " or "Nicaragua" or "Niger" or "Nigeria" or "North Korea" or "Oman" or "Pakistan" or "Palau " or "Palestine" or "Panama" or "Papua New Guinea " or "Paraguay" or "Peru" or "Philippines" or "Puerto Rico" or "Qatar" or "Republic of the Congo" or "Russia" or "Rwanda" or "Saint Kitts" or "Saint Lucia" or "Saint Vincent " or "Samoa Solomon Islands" or "Sao Tome" or "Saudi Arabia" or "Senegal" or "Seychelles" or "Sierra Leone" or "Singapore" or "Somalia" or "South Africa" or "South America" or "South Korea" or "South Sudan" or "Sri Lanka" or "Sudan" or "Sulawesi" or "Sumbawa" or "Suriname" or "Swaziland" or "Syria" or "Taiwan" or "Tajikistan" or "Tanzania" or "Tchad" or "Thailand" or "The Gambia" or "Timor" or "Tobago" or "Togo" or "Tonga" or "Trinidad " or "Tunisia" or "Turkey" or "Turkmenistan" or "Turks and Caicos" or "Tuvalu" or "Uganda" or "United Arab Emirates" or "Uruguay" or "Uzbekistan" or "Vanuatu" or "Venezuela" or "Vietnam" or "Western Sahara" or "Yemen" or "Zaire" or "Zambia" or "Zimbabwe") not ("Australia" or "Canada" or "England" or "Europe" or "Ireland" or "New Zealand" or "North America" or "Scotland" or "United Kingdom" or "US" or "Wales")).lo. | 232535 |
| 9 | 7 not 8 | 479 |
| 10 | "sustainable development goal*".ti,ab,id. | 167 |
| 11 | 9 not 10 | 468 |
| 12 | "Conservation (Ecological Behavior)"/ or *Climate Change/ or *Ecology/ or *Environmental Effects/ or *Global Warming/ or *Natural Resources/ or *Pollution/ | 13080 |
| 13 | 11 not 12 | 458 |
| 14 | (1* or 2* or 3* or 4* or 5* or 6* or 7* or 8* or 9*).pm. | 1590960 |
| 15 | 13 not 14 | 230 |
| 16 | remove duplicates from 15 | 230 |

CINAHL Plus with Full Text

| # | Query | Results |
| --- | --- | --- |
| S1 | (MH "Community Health Nursing") OR (MH "Community Health Services") OR (MH "Health Education") OR (MH "Health Policy") OR (MH "Health Promotion") OR (MH "Preventive Health Care") OR (MH "Public Health Administration") OR (MH "Public Health") OR (MH "United States Public Health Service") OR TI("health authorit*" OR "health department*" OR "health education" OR "health policies" OR "health policy" OR "health program*" OR "health promot*" OR "health unit*" OR "population health" OR "public health" OR (communit* N3 health*) OR (disease* N3 prevent*) OR prevention OR preventive) OR SU("health authorit*" OR "health department*" OR "health education" OR "health policies" OR "health policy" OR "health program*" OR "health promot*" OR "health unit*" OR "population health" OR "public health" OR (communit* N3 health*) OR (disease* N3 prevent*) OR "primary prevention" OR preventive) | 377,343 |
| S2 | (MH "Coalition") OR (MH "Community Programs") OR (MH "Government Programs") OR (MH "Health and Welfare Planning") OR (MH "Immunization Programs") OR (MH "National Health Programs") OR (MH "Needle Exchange Programs") OR (MH "Pilot Studies") OR (MH "Planning Techniques") OR (MH "Program Development") OR (MH "Program Evaluation") OR (MH "Program Implementation") OR (MH "Program Planning") OR (MH "Smoking Cessation Programs") OR (MH "State Health Plans") OR (MH "Strategic Planning") OR (MH "Weight Reduction Programs") OR TI("action plan*" OR "community challenge*" OR "community partnership*" OR "health plan*" OR "sustainable health promotion action" OR "tak* action" OR "task force*" OR (health N3 action*) OR approach OR approaches OR campaign* OR coalition* OR implement* OR initiative* OR innovation* OR pilot* OR policies OR policy OR program* OR project*) OR SU("action plan*" OR "community challenge*" OR "community partnership*" OR "health plan*" OR "sustainable health promotion action" OR "tak* action" OR "task force*" OR (health N3 action*) OR approach OR approaches OR campaign* OR coalition* OR implement* OR initiative* OR innovation* OR pilot* OR policies OR policy OR program* OR project*) OR program OR programs OR programme OR programmes | 655,654 |
| S3 | TI(sustainab* or sustainment or routinis* or routiniz* or "long term implementation" or "long term effectiveness" or "long lasting" or "voltage drop*" or "program drift*" or ((maintain* or continue* or continuing or continuation or last* or durab* or persist* or sustain*) N5 (change* or benefit* or effective* or outcome* or behav* or fund* or program* or service* or resources or "long term" or "over time" or "extended period of time")) or endgame) OR SU(sustainab* or sustainment or routinis* or routiniz* or "long term implementation" or "long term effectiveness" or "long lasting" or "voltage drop*" or "program drift*" or ((maintain* or continue* or continuing or continuation or last* or durab* or persist* or sustain*) N5 (change* or benefit* or effective* or outcome* or behav* or fund* or program* or service* or resources or "long term" or "over time" or "extended period of time")) or endgame) | 8,153 |
| S4 | TI sustain* AND AB sustain* | 3,576 |
| S5 | S1 AND S2 AND (S3 OR S4) | 884 |
| S6 | S5 AND LA English | 873 |
| S7 | S6 AND DT 19980101-20181231 | 842 |
| S8 | ( (MH "Africa+") OR (MH "Asia+") OR (MH "South America+") OR (MH "West Indies+") OR (MH "Central America+") OR (MH "Latin America") OR (MH "Atlantic Islands+") OR (MH "Developing Countries") OR (MH "Indian Ocean Islands+") OR (MH "Micronesia+") OR (MH "Melanesia+") OR (MH "Polynesia+") ) NOT ( (MH "North America") OR (MH "Canada+") OR (MH "United States+") OR (MH "Europe+") OR (MH "Developed Countries") OR (MH "Australia+") OR (MH "New Zealand") ) | 300,391 |
| S9 | S7 NOT S8 | 710 |
| S10 | "sustainable development goal*" | 593 |
| S11 | S9 NOT S10 | 682 |
| S12 | (MM "Environmental Health") OR (MM "Climate Change") OR (MM "Greenhouse Effect") OR (MM "Envinonmental Pollution") OR (MM "Ecosystem") OR (MM "Conservation of Natural Resources") | 6,450 |
| S13 | S11 NOT S12 | 632 |
| S14 | S13 AND MX Y | 245 |

SocINDEX with Full text

| # | Query | Results |
| --- | --- | --- |
| S1 | "health authorit*" OR "health department*" OR "health education" OR "health policies" OR "health policy" OR "health program*" OR "health promot*" OR "health unit*" OR "population health" OR "public health" OR (communit* N3 health*) OR (disease* N3 prevent*) OR prevention OR preventive | 226,648 |
| S2 | TI("action plan*" OR "community challenge*" OR "community partnership*" OR "health plan*" OR "sustainable health promotion action" OR "tak* action" OR "task force*" OR (health N3 action*) OR approach OR approaches OR campaign* OR coalition* OR implement* OR initiative* OR innovation* OR pilot* OR policies OR policy OR program* OR project*) OR SU("action plan*" OR "community challenge*" OR "community partnership*" OR "health plan*" OR "sustainable health promotion action" OR "tak* action" OR "task force*" OR (health N3 action*) OR approach OR approaches OR campaign* OR coalition* OR implement* OR initiative* OR innovation* OR pilot* OR policies OR policy OR program* OR project*) OR program OR programs OR programme OR programmes | 484,248 |
| S3 | TI(sustainab* or sustainment or routinis* or routiniz* or "long term implementation" or "long term effectiveness" or "long lasting" or "voltage drop*" or "program drift*" or ((maintain* or continue* or continuing or continuation or last* or durab* or persist* or sustain*) N5 (change* or benefit* or effective* or outcome* or behav* or fund* or program* or service* or resources or "long term" or "over time" or "extended period of time")) or endgame) OR SU(sustainab* or sustainment or routinis* or routiniz* or "long term implementation" or "long term effectiveness" or "long lasting" or "voltage drop*" or "program drift*" or ((maintain* or continue* or continuing or continuation or last* or durab* or persist* or sustain*) N5 (change* or benefit* or effective* or outcome* or behav* or fund* or program* or service* or resources or "long term" or "over time" or "extended period of time")) or endgame) | 8,117 |
| S4 | TI sustain* AND AB sustain* | 3,866 |
| S5 | S1 AND S2 AND (S3 OR S4) | 646 |
| S6 | S5 AND LA English | 590 |
| S7 | S6 AND DT 19980101-20181231 | 506 |
| S8 | GE ( (("Afghanistan" or "Africa" or "Algeria" or "Angola" or "Antigua" or "Argentina" or "Armenia" or "Asia" or "Azerbaijan" or "Bahamas" or "Bahrain" or "Bangladesh" or "Barbados" or "Barbuda" or "Belize" or "Benin" or "Bhutan" or "Bolivia" or "Botswana" or "Brazil" or "Brunei" or "Burkina Faso" or "Burma" or "Burundi" or "Cambodia" or "Cameroon" or "Cape Verde" or "Central African Republic" or "Central America" or "Chad" or "Chile" or "China" or "Colombia" or "Comoros" or "Costa Rica" or "Côte d'Ivoire" or "Cuba" or "Curacao" or "Cyprus" or "Democratic Republic of the Congo" or "Djibouti" or "Dominica" or "Dominican Republic" or "East Timor" or "Ecuador" or "Egypt" or "El Salvador" or "Equatorial Guinea" or "Eritrea" or "Ethiopia" or "Fiji " or "Flores" or "French Guiana" or "Gabon" or "Georgia" or "Ghana" or "Grenada" or "Guatemala" or "Guinea" or "Guinea-Bissau" or "Guyana" or "Haiti" or "Honduras" or "India" or "Indonesia" or "Iran" or "Iraq" or "Israel" or "Ivory Coast" or "Jamaica" or "Japan" or "Jerusalem" or "Jordan" or "Kazakhstan" or "Kenya" or "Kiribati " or "Kuwait" or "Kyrgyzstan" or "Laos" or "Lebanon" or "Lesotho" or "Liberia" or "Libya" or "Lombok" or "Madagascar" or "Malawi" or "Malaysia" or "Maldives" or "Mali" or "Marshall Islands " or "Mauritania" or "Mauritius" or "Melanesia" or "Mexico" or "Micronesia " or "Mongolia" or "Morocco" or "Mozambique" or "Myanmar " or "Namibia" or "Nauru " or "Nepal" or "New Caledonia" or "New Guinea" or "New Zealand " or "Nicaragua" or "Niger" or "Nigeria" or "North Korea" or "Oman" or "Pakistan" or "Palau " or "Palestine" or "Panama" or "Papua New Guinea " or "Paraguay" or "Peru" or "Philippines" or "Puerto Rico" or "Qatar" or "Republic of the Congo" or "Russia" or "Rwanda" or "Saint Kitts" or "Saint Lucia" or "Saint Vincent " or "Samoa Solomon Islands" or "Sao Tome" or "Saudi Arabia" or "Senegal" or "Seychelles" or "Sierra Leone" or "Singapore" or "Somalia" or "South Africa" or "South America" or "South Korea" or "South Sudan" or "Sri Lanka" or "Sudan" or "Sulawesi" or "Sumbawa" or "Suriname" or "Swaziland" or "Syria" or "Taiwan" or "Tajikistan" or "Tanzania" or "Tchad" or "Thailand" or "The Gambia" or "Timor" or "Tobago" or "Togo" or "Tonga" or "Trinidad " or "Tunisia" or "Turkey" or "Turkmenistan" or "Turks and Caicos" or "Tuvalu" or "Uganda" or "United Arab Emirates" or "Uruguay" or "Uzbekistan" or "Vanuatu" or "Venezuela" or "Vietnam" or "Western Sahara" or "Yemen" or "Zaire" or "Zambia" or "Zimbabwe") not ("Australia" or "Canada" or "England" or "Europe" or "Ireland" or "New Zealand" or "North America" or "Scotland" or "United Kingdom" or "US" or "United States" or "Wales")) ) OR TI ( (("Afghanistan" or "Africa" or "Algeria" or "Angola" or "Antigua" or "Argentina" or "Armenia" or "Asia" or "Azerbaijan" or "Bahamas" or "Bahrain" or "Bangladesh" or "Barbados" or "Barbuda" or "Belize" or "Benin" or "Bhutan" or "Bolivia" or "Botswana" or "Brazil" or "Brunei" or "Burkina Faso" or "Burma" or "Burundi" or "Cambodia" or "Cameroon" or "Cape Verde" or "Central African Republic" or "Central America" or "Chad" or "Chile" or "China" or "Colombia" or "Comoros" or "Costa Rica" or "Côte d'Ivoire" or "Cuba" or "Curacao" or "Cyprus" or "Democratic Republic of the Congo" or "Djibouti" or "Dominica" or "Dominican Republic" or "East Timor" or "Ecuador" or "Egypt" or "El Salvador" or "Equatorial Guinea" or "Eritrea" or "Ethiopia" or "Fiji " or "Flores" or "French Guiana" or "Gabon" or "Georgia" or "Ghana" or "Grenada" or "Guatemala" or "Guinea" or "Guinea-Bissau" or "Guyana" or "Haiti" or "Honduras" or "India" or "Indonesia" or "Iran" or "Iraq" or "Israel" or "Ivory Coast" or "Jamaica" or "Japan" or "Jerusalem" or "Jordan" or "Kazakhstan" or "Kenya" or "Kiribati " or "Kuwait" or "Kyrgyzstan" or "Laos" or "Lebanon" or "Lesotho" or "Liberia" or "Libya" or "Lombok" or "Madagascar" or "Malawi" or "Malaysia" or "Maldives" or "Mali" or "Marshall Islands " or "Mauritania" or "Mauritius" or "Melanesia" or "Mexico" or "Micronesia " or "Mongolia" or "Morocco" or "Mozambique" or "Myanmar " or "Namibia" or "Nauru " or "Nepal" or "New Caledonia" or "New Guinea" or "New Zealand " or "Nicaragua" or "Niger" or "Nigeria" or "North Korea" or "Oman" or "Pakistan" or "Palau " or "Palestine" or "Panama" or "Papua New Guinea " or "Paraguay" or "Peru" or "Philippines" or "Puerto Rico" or "Qatar" or "Republic of the Congo" or "Russia" or "Rwanda" or "Saint Kitts" or "Saint Lucia" or "Saint Vincent " or "Samoa Solomon Islands" or "Sao Tome" or "Saudi Arabia" or "Senegal" or "Seychelles" or "Sierra Leone" or "Singapore" or "Somalia" or "South Africa" or "South America" or "South Korea" or "South Sudan" or "Sri Lanka" or "Sudan" or "Sulawesi" or "Sumbawa" or "Suriname" or "Swaziland" or "Syria" or "Taiwan" or "Tajikistan" or "Tanzania" or "Tchad" or "Thailand" or "The Gambia" or "Timor" or "Tobago" or "Togo" or "Tonga" or "Trinidad " or "Tunisia" or "Turkey" or "Turkmenistan" or "Turks and Caicos" or "Tuvalu" or "Uganda" or "United Arab Emirates" or "Uruguay" or "Uzbekistan" or "Vanuatu" or "Venezuela" or "Vietnam" or "Western Sahara" or "Yemen" or "Zaire" or "Zambia" or "Zimbabwe") not ("Australia" or "Canada" or "England" or "Europe" or "Ireland" or "New Zealand" or "North America" or "Scotland" or "United Kingdom" or "US" or "United States" or "Wales")) ) | 223,602 |
| S9 | S7 NOT S8 | 418 |
| S10 | "sustainable development goal*" | 177 |
| S11 | S9 NOT S10 | 399 |
| S12 | SU (environmental or ecolog* or "climate change" or pollution) | 26,908 |
| S13 | S11 NOT S12 | 291 |

Scopus-Name of database (long)

| # | Query | Results |
| --- | --- | --- |
|  | ( TITLE-ABS-KEY ( "health authorit*" OR "health department*" OR "health education" OR "health policies" OR "health policy" OR "health program*" OR "health promot*" OR "health unit*" OR "population health" OR "public health" OR ( communit* W/3 health* ) OR ( disease* W/3 prevent* ) OR prevention OR preventive ) AND ( TITLE ( "action plan*" OR "community challenge*" OR "community partnership*" OR "health plan*" OR "sustainable health promotion action" OR "tak* action" OR "task force*" OR ( health W/3 action* ) OR approach OR approaches OR campaign* OR coalition* OR implement* OR initiative* OR innovation* OR pilot* OR policies OR policy OR program* OR project ) OR KEY ( "action plan*" OR "community challenge*" OR "community partnership*" OR "health plan*" OR "sustainable health promotion action" OR "tak* action" OR "task force*" OR ( health W/3 action* ) OR approach OR approaches OR campaign* OR coalition* OR implement* OR initiative* OR innovation* OR pilot* OR policies OR policy OR program* OR project ) OR TITLE-ABS-KEY ( program OR programs OR programme OR programmes ) ) AND ( TITLE ( sustainab* OR sustainment OR routinis* OR routiniz* OR "long term implementation" OR "long term effectiveness" OR "long lasting" OR "voltage drop*" OR "program drift*" OR ( ( maintain* OR continue* OR continuing OR continuation OR last* OR durab* OR persist* OR sustain* ) W/5 ( change* OR benefit* OR effective* OR outcome* OR behav* OR fund* OR program* OR service* OR resources OR "long term" OR "over time" OR "extended period of time" ) ) OR endgame ) OR KEY ( sustainab* OR sustainment OR routinis* OR routiniz* OR "long term implementation" OR "long term effectiveness" OR "long lasting" OR "voltage drop*" OR "program drift*" OR ( ( maintain* OR continue* OR continuing OR continuation OR last* OR durab* OR persist* OR sustain* ) W/5 ( change* OR benefit* OR effective* OR outcome* OR behav* OR fund* OR program* OR service* OR resources OR "long term" OR "over time" OR "extended period of time" ) ) OR endgame ) OR ( TITLE ( sustain* ) AND ABS ( sustain* ) ) ) AND LANGUAGE ( english ) AND PUBYEAR AFT 1997 AND SUBJAREA ( soci AND NOT envi ) AND AFFILCOUNTRY ( "Undefined" OR "United States" OR "Canada" OR "Australia" OR "New Zealand" OR "United Kingdom" OR "England" OR "Ireland" OR "Scotland" OR "Wales" ) ) AND NOT INDEX ( medline ) | 239 |

**Appendix B**

Grey literature repositories

The New York Academy of Medicine Grey Literature Report**-**www.greylit.org

| Search Query | Date Searched | # Records Retrieved |
| --- | --- | --- |
| sustainability | 06/29/2018 | 137 |

Web searches (custom search engines)

Custom Search Engine for Canadian Public Health Information <http://ophla.ca/customsearchcanada.htm>

| Search Strategy | Date Searched | # Records Reviewed |
| --- | --- | --- |
| program sustainability "public health" OR "health promotion" -environmental -"sustainable development" | 06/30/2018 | 50 |
| program sustainability "community health" OR "population health" OR "disease prevention" OR "health policy" OR "health education" -environmental -"sustainable development" | 06/30/2018 | 50 |
| sustainability institutionalization OR routinization OR "voltage drop" OR "program drift" "public health" OR "health promotion" OR "community health" OR "population health" OR "disease prevention" OR "health policy" OR "health education" | 06/30/2018 | 50 |
| program sustainability outcomes OR "health effects" OR effectiveness OR benefits OR "social change" "public health" OR "health promotion" OR "community health" OR "population health" OR "disease prevention" OR "health policy" OR "health education" | 06/30/2018 | 50 |
| program sustainability funding OR financial "public health" OR "health promotion" OR "community health" OR "population health" OR "disease prevention" OR "health policy" OR "health education" | 06/30/2018 | 50 |
| "public health" OR "health promotion" "sustainability of program" OR "sustainability of programs" OR "sustainability of the program" OR "sustainability of the programs" OR "sustainable program" OR "sustainable programs" OR "program sustainability" | 06/30/2018 | 50 |

The following sites/domains are searched by this custom search engine:

- Health Canada | canada.ca/en/health-Canada
- Public Health Agency of Canada | canada.ca/en/public-health
- Canadian Best Practices Portal | cbpp-pcpe.phac-aspc.gc.ca
- Alberta
- Alberta Health | health.alberta.ca
- Alberta Health Services | albertahealthservices.ca
- British Columbia Ministry of Health | gov.bc.ca
- British Columbia Provincial Health Services Authority | phsa.ca
- British Columbia First Nations Health Authority | fnha.ca
- British Columbia Centre for Disease Control | bccdc.ca
- Fraser Health | fraserhealth.ca
- Interior Health | interiorhealth.ca
- Island Health | viha.ca
- Northern Health | northernhealth.ca
- Vancouver Coastal Health | vch.ca
- Manitoba Health | gov.mb.ca
- Interlake-Eastern Regional Health Authority | ierha.ca
- Northern Regional Health Authority | nrha.ca
- Southern Health/Santé Sud | southernhealth.ca
- Prairie Mountain Health | prairiemountainhealth.ca
- Winnipeg Regional Health Authority | wrha.mb.ca
- New Brunswick Department of Health | gnb.ca
- Horizon Health Network | horizonnb.ca
- Vitalité Health Network | vitalitenb.ca
- Newfoundland Department of Health and Community Services | health.gov.nl.ca
- Eastern Health | easternhealth.ca
- Central Health | centralhealth.nl.ca
- Western Health | westernhealth.nl.ca
- Labrador-Grenfell Health | lghealth.ca
- Nova Scotia Department of Health and Wellness | novascotia.ca/dhw
- Nova Scotia Health Authority | nshealth.ca
- Nunavut Department of Health | gov.nu.ca
- Northwest Territories Department of Health and Social Services | hss.gov.nt.ca
- Beaufort-Delta Health and Social Services Authority | bdhssa.hss.gov.nt.ca
- Dehcho Health and Social Services Authority | dhssa.hss.gov.nt.ca
- Fort Smith Health and Social Services Authority | fshssa.hss.gov.nt.ca
- Hay River Health and Social Services Authority | hrhssa.org
- Sahtu Health and Social Services Authority | shssa.hss.gov.nt.ca
- Stanton Territorial Health Authority | stha.hss.gov.nt.ca
- Tłı̨chǫ Community Services Agency | tlicho.ca
- Yellowknife Health and Social Services Authority | yhssa.hss.gov.nt.ca
- Ontario Ministry of Health and Long-Term Care | health.gov.on.ca
- Public Health Ontario | publichealthontario.ca
- Algoma Public Health | algomapublichealth.com
- Brant County Health Unit | bchu.org
- Chatham-Kent Public Health Unit | ckphu.com
- Durham Region Public Health | durham.ca
- Eastern Ontario Health Unit | eohu.ca
- Elgin St. Thomas Public Health | elginhealth.on.ca
- Grey Bruce Health Unit | publichealthgreybruce.on.ca
- Haldimand-Norfolk Health Unit | hnhu.org
- Haliburton, Kawartha, Pine Ridge District Health Unit | hkpr.on.ca
- Halton Region Public Health | halton.ca
- Hamilton Public Health | hamilton.ca
- Hastings Prince Edward Public Health | hpepublichealth.ca
- Huron County Health Unit | huronhealthunit.ca
- KFL&A Public Health | kflaph.ca
- Lambton Public Health | lambtonhealth.on.ca
- Leeds, Grenville and Lanark District Health Unit | healthunit.org
- Middlesex-London Health Unit | healthunit.com
- Niagara Region Public Health | niagararegion.ca
- North Bay Parry Sound District Health Unit | myhealthunit.ca
- Northwestern Health Unit | nwhu.on.ca
- Ottawa Public Health | ottawapublichealth.ca
- Oxford County Public Health | oxfordcounty.ca
- Peel Public Health | peelregion.ca
- Perth District Health Unit | pdhu.on.ca
- Peterborough Public Health | peterboroughpublichealth.ca
- Porcupine Health Unit | porcupinehu.on.ca
- Renfrew County and District Health Unit | rcdhu.com
- Simcoe Muskoka District Health Unit | simcoemuskokahealth.org
- Public Health Sudbury & Districts | phsd.ca
- Thunder Bay District Health Unit | tbdhu.com
- Timiskaming Health Unit | timiskaminghu.com
- Toronto Public Health | toronto.ca
- Region of Waterloo Public Health | regionofwaterloo.ca
- Wellington-Dufferin-Guelph Public Health | wdgpublichealth.ca
- Windsor-Essex County Health Unit | wechu.org
- York Region Public Health | york.ca
- Health PEI | princeedwardisland.ca
- Institut national de santé publique du Québec | inspq.qc.ca
- Ministère de la Santé et des Services sociaux | msss.gouv.qc.ca
- Nunavik Regional Board of Health and Social Services | nrbhss.gouv.qc.ca
- Cree Board of Health and Social Services of James Bay | creehealth.org
- Saskatchewan Health Authority | saskhealthauthority.ca
- Saskatchewan Ministry of Health | saskatchewan.ca
- Cypress Regional Health Authority | cypresshealth.ca
- Five Hills Regional Health Authority | fhhr.ca
- Heartland Regional Health Authority | hrha.sk.ca
- Keewatin Yatthé Regional Health Authority | kyrha.ca
- Kelsey Trail Regional Health Authority | kelseytrailhealth.ca
- Mamawetan Churchill River Health Region | mcrhealth.ca
- Prairie North Health Region | pnrha.ca
- Prince Albert Parkland Health Region | paphr.ca
- Regina Qu'Appelle Health Region | rqhealth.ca
- Saskatoon Health Region | saskatoonhealthregion.ca
- Sun Country Health Region | suncountry.sk.ca
- Sunrise Health Region | sunrisehealthregion.sk.ca
- Department of Health and Social Services | hss.gov.yk.ca
- National Collaborating Centres for Public Health | nccph.ca
- National Collaborating Centre for Aboriginal Health | nccah-ccnsa.ca
- National Collaborating Centre for Determinants of Health | nccdh.ca
- National Collaborating Centre for Environmental Health | ncceh.ca
- National Collaborating Centre for Healthy Public Policy | ncchpp.ca
- National Collaborating Centre for Infectious Diseases | nccid.ca
- National Collaborating Centre for Methods and Tools | nccmt.ca

International public health (outside of Canada) https://cse.google.com/cse/publicurl?cx=010268328710930794660:bclwerxwfeq

| Search Strategy | Date Searched | # Records Reviewed |
| --- | --- | --- |
| program sustainability "public health" OR "health promotion" -environmental -"sustainable development" | 06/30/2018 | 50 |
| program sustainability "community health" OR "population health" OR "disease prevention" OR "health policy" OR "health education" -environmental -"sustainable development" | 06/30/2018 | 50 |
| sustainability institutionalization OR routinization OR "voltage drop" OR "program drift" "public health" OR "health promotion" OR "community health" OR "population health" OR "disease prevention" OR "health policy" OR "health education" | 06/30/2018 | 50 |
| program sustainability outcomes OR "health effects" OR effectiveness OR benefits OR "social change" "public health" OR "health promotion" OR "community health" OR "population health" OR "disease prevention" OR "health policy" OR "health education" | 06/30/2018 | 50 |
| program sustainability funding OR financial "public health" OR "health promotion" OR "community health" OR "population health" OR "disease prevention" OR "health policy" OR "health education" | 06/30/2018 | 50 |
| "public health" OR "health promotion" "sustainability of program" OR "sustainability of programs" OR "sustainability of the program" OR "sustainability of the programs" OR "sustainable program" OR "sustainable programs" OR "program sustainability" | 06/30/2018 | 50 |

The following sites/domains are searched by this custom search engine:

- African Union | au.int
- Agency for Healthcare Research and Quality | ahrq.gov
- Association of Medical Microbiology and Infectious Disease Canada (AMMI Canada) | ammi.ca
- Association of State and Territorial Health Officials |astho.org
- Australian Capital Territory | act.gov.au
- Australian government | australia.gov.au
- CDC (Centers for Disease Control and Prevention) | cdc.gov
- CIDRAP (Center for Infectious Disease Research and Policy) | cidrap.umn.edu
- ECDC (European Centre for Disease Prevention and Control) | ecdc.europa.eu
- European Commission public health | ec.europa.eu/health
- European Public Health Association | eupha.org
- Health and Medicine Division of National Academies Sciences Engineering Medicine | nationalacademies.org/hmd
- Health Protection Scotland | hps.scot.nhs.uk
- International Union for Health Promotion and Research | iuhpe.org
- NACCHO: National Association of County and City Health Officials | naccho.org
- National Centre for Immunisation Research & Surveillance (Australia) | ncirs.edu.au
- New South Wales | nws.gov.au
- New Zealand government | govt.nz
- NHS | nhs.uk
- NICE : National Institute for Health and Care Excellence | nice.org.uk
- Northern Territory | nt.gov.au
- Pan American Health Organization | paho.org
- Public Health Agency Northern Ireland | publichealth.hscni.net
- Public Health Association of New Zealand | pha.org.nz
- Public Health Foundation | phf.org
- Public Health Wales | wales.nhs.uk
- Queensland | qld.gov.au
- ScotPHN (Scottish Public Health Network) | scotphn.net
- ScotPHO (Scottish Public Health Observatory) | scotpho.org.uk
- South Australia | sa.gov.au
- Tasmania | tas.gov.au
- United Kingdom Government (includes Public Health England) | gov.uk
- Victoria | vic.gov.au
- Western Australia | wa.gov.au
- World Health Organization | www.who.int

Web searches (general)

Google Canada https://www.google.ca/

| Search Strategy | Date Searched | # Records Reviewed |
| --- | --- | --- |
| program sustainability "public health" OR "health promotion" -environmental -"sustainable development" | 06/30/2018 | 100 |
| program sustainability "community health" OR "population health" OR "disease prevention" OR "health policy" OR "health education" -environmental -"sustainable development" | 06/30/2018 | 100 |
| sustainability institutionalization OR routinization OR "voltage drop" OR "program drift" "public health" OR "health promotion" OR "community health" OR "population health" OR "disease prevention" OR "health policy" OR "health education" | 06/30/2018 | 100 |
| program sustainability outcomes OR "health effects" OR effectiveness OR benefits OR "social change" "public health" OR "health promotion" OR "community health" OR "population health" OR "disease prevention" OR "health policy" OR "health education" | 06/30/2018 | 100 |
| program sustainability funding OR financial "public health" OR "health promotion" OR "community health" OR "population health" OR "disease prevention" OR "health policy" OR "health education" | 06/30/2018 | 100 |
| "public health" OR "health promotion" "sustainability of program" OR "sustainability of programs" OR "sustainability of the program" OR "sustainability of the programs" OR "sustainable program" OR "sustainable programs" OR "program sustainability" | 06/30/2018 | 100 |
