# Supplementary material for: Sustainable by design: a systematic review of factors for health promotion program sustainability
Source: BMC Public Health. 2020 Jun 19;20:964. doi: 10.1186/s12889-020-09091-9 (PMC7304137; doi:10.1186/s12889-020-09091-9)
Supplement: Supplementary file 2 — Additional file 2: Appendix C. Results of quality assessment of the included 16 papers with final ratings. [file 12889_2020_9091_MOESM2_ESM.docx]

# Appendix C

**Health Evidence Quality Assessment Tool for Systematic Reviews and Meta-analyses**

| **Criterion** | **Hodge 2016** | **Hanson 2005** | **Johnson 2005** | **Scheirer 2005** | **Whelan 2018** | **Nelson 2007** |
| --- | --- | --- | --- | --- | --- | --- |
|  | | |  |  |  |  |
| 1. Did the authors have a clearly focused question [population, intervention (strategy), and outcomes(s)]? (1 point) | Yes | Yes | Yes | Yes | Yes | Yes |
| 1. Were appropriate inclusion criteria used to select primary studies? (1 point) | Yes | No | No | Yes | Yes | No |
| 1. Did the authors describe a search strategy that was comprehensive? (1 point) | Yes | No | No | Yes | No | No |
| 1. Did search strategy cover an adequate number of years? (1 point) | Yes | No | No | Yes | Yes | No |
| 1. Did the authors describe the level of evidence in the primary studies included in the review? (1 point) | Yes | No | No | Yes | Yes | No |
| 1. Did the review assess the methodological quality of the primary studies? (1 point) | No | No | No | No | Yes | No |
| 1. Are the results of the review transparent? (1 point) | No | No | No | No | No | No |
| 1. Was it appropriate to combine the findings of results across studies? (1 point) | Yes | No | No | Yes | Yes | No |
| 1. Were appropriate methods used for combining or comparing results across studies? (1 point) | No | No | No | No | No | No |
| 1. Do the data support the author's interpretation? (1 point) | No | No | No | Yes | Yes | No |
| **Total Score** | **6** | **1** | **1** | **7** | **7** | **1** |
| **Rank** | **Moderate** | **Weak** | **Weak** | **Moderate** | **Moderate** | **Weak** |

**Note: Total score calculation (total number of points obtained); Highest possible score is 10. Reviews with a score of 8 or higher were rated strong, a score between 5-7 as moderate, and a score of 4 or less as weak.**

**Criteria for appraising qualitative research studies (CASP Tool)**

| **Criterion** | **Ammerman 2002*** | **Bumbarger and Perkins 2008** | **Casey 2009** | **Paine-Andrews 2000** | **Sadof 2006** | **Schell 2013** | **Wisener 2017** | **Garst 2017** | **Harris and Sandor 2013*** | **Swerrisen and Crisp** | **Carstensen 2018** |
| --- | --- | --- | --- | --- | --- | --- | --- | --- | --- | --- | --- |
| **Screening questions (2 points)** | | | | |  |  |  |  |  |  |  |
| 1. Was there a clear statement of the aims of the research? (1 point) | Yes | Yes | Yes | Yes | Yes | Yes | Yes | Yes | Yes | Yes | Yes |
| 1. Is a qualitative methodology appropriate? (1 point) | Yes | No | Yes | Yes | Yes | Yes | Yes | Yes | Yes | No | Yes |
| **IS IT WORTH CONTINUING?** | Yes | No | Yes | Yes | Yes | Yes | Yes | Yes | Yes | No | Yes |
| **Detailed questions (8 points)** | | | | |  |  |  |  |  |  |  |
| 1. Was the research design appropriate to address the aims of the research? (1 point) | Yes | - | Yes | Yes | Yes | Yes | Yes | Yes | Yes | - | Yes |
| 1. Was the recruitment strategy appropriate to the aims of the research? (1 point) | Yes | - | Yes | Yes | Yes | Yes | Yes | Yes | Yes | - | Yes |
| 1. Was the data collected in a way that addressed the research issue? (1 point) | Yes | - | Yes | Yes | Yes | Yes | Yes | Yes | Yes | - | Yes |
| 1. Has the relationship between researcher and participants been adequately considered? (1 point) | No | - | No | No | Yes | N/A | Yes | No | No | - | No |
| 1. Have ethical issues been taken into consideration? (1 point) | Yes | - | Yes | No | Yes | N/A | Yes | No | Yes | - | Yes |
| 1. Was the data analysis sufficiently rigorous? (1 point) | No | - | Yes | Yes | Yes | Yes | Yes | Yes | Yes | - | Yes |
| 1. Is there a clear statement of findings? (1 point) | Yes | - | Yes | Yes | Yes | Yes | Yes | Yes | Yes | - | Yes |
| 1. Is the research valuable? (1 point) | No | - | Yes | Yes | Yes | Yes | Yes | Yes | Yes | - | Yes |
| **Total Score** | **7** | **1** | **9** | **8** | **10** | **8** | **10** | **8** | **9** | **1** | **9** |
| **Rank** | **Moderate** | **Weak** | **Strong** | **Strong** | **Strong** | **Strong** | **Strong** | **Strong** | **Strong** | **Weak** | **Strong** |

**Note: Total score calculation (total number of points obtained). Highest possible score is 10. Specific prompts were provided for each criterion to assist in rating. Articles and papers with a score of 8 or higher were rated strong, a score between 5-7 as moderate, and a score of 4 or less as weak.**

***Study was later excluded as it was determined to have met exclusion criteria**

**Quality Assessment Tool for Cohort studies (Newcastle-Ottawa Quality Assessment Form for Cohort Studies)**

| **Criterion** | **Curry 2006*** |
| --- | --- |
| **Selection** |  |
| 1. Representativeness of the sample 2. Truly representative of the average in the target population (one star) 3. Somewhat representative (one star) 4. Selected group of users 5. No description of the derivation of the cohort | b (1 star) |
| 1. Sample size 2. Justified and satisfactory (one star) 3. Not justified | b (0 star) |
| 1. Non-respondents 2. comparability between respondents and non-respondents characteristics established, and the response rate is satisfactory (one star) 3. the response rate is unsatisfactory, or the comparability between respondents and non-respondents is unsatisfactory 4. no description of the response rate or the characteristics of the responders and the non-responders | a (1 star) |
| 1. Ascertainment of the exposure 2. Validated measurement tool (two stars) 3. Non-validated measurement tool, but the tool is available or described (one star) 4. No description of the measurement tool | b (1 star) |
| 1. The subjects in different outcomes groups are comparable, based on the study design or analysis. Confounding factors are controlled. 2. The study controls for the most important factors (one star) 3. The study controls for any additional factor (one star) | (0 star) |
| 1. Assessment of outcome   a) Independent blind assessment (two star)  b) Record linkage (two star)  c) Self report (one star)  d) No description | c (1 star) |
| 1. Statistical test 2. The statistical test used to analyze the data is clearly described and appropriate, and the measurement of the association is presented, including confidence intervals and the probability level (one star) 3. The statistical test is not appropriate, not described or incomplete. | 1 (1 star) |
| **Total Score** | **5** |
| **Rank** | **Moderate** |

**Note: 0-4 is weak, 5-7 is moderate, and 8-10 is strong.**

***Study was later excluded as it was determined to have meet exclusion criteria**

**Grey Literature Appraisal Tool (Caldwell 2011; Bergeron 2017)**

| **Criterion** | **Elsworth 2005** | **Hill 2011** | **Altarum Institute 2009** | **Office of Adolescent**  **Health 2014** | **Office of Adolescent Health 2017** | **Scheirer 2014** | **Scheirer 2012** | **Education Development Centre 2017** | **Buck 2015** | **Georgia Health Policy Centre 2011** | **National Centre for Mental Health Promotion and Youth Violence Prevention** |
| --- | --- | --- | --- | --- | --- | --- | --- | --- | --- | --- | --- |
| **Screening questions (no points)** | | | | | |  |  |  |  |  |  |
| 1. The methods used to develop the product are clearly stated | Yes | Yes | Yes | No | No | No | No | No | No | No | No |
| 1. 2. Methods could be determined (e.g., through searching additional related sources) within 2 clicks | n/a | Yes | n/a | Yes | Yes | No | No | No | No | No | No |
| **IS IT WORTH CONTINUING?** | Yes | Yes | Yes | Yes | Yes | No | No | No | No | No | No |
| **Quality assessment questions (1 point)** | | | | | |  |  |  |  |  |  |
| 1. Are the authors credible? (e.g., document is published by a university, government agency, author has other published work) | Yes | Yes | Yes | Yes | Yes | - | - | - | - | - | - |
| 2. Is the rational for the resource clearly identified? (e.g., a purpose statement or research question) | Yes | Yes | Yes | Yes | Yes | - | - | - | - | - | - |
| 3. Is the methodology clear and transparent? | Yes | Yes | Yes | Yes | Yes | - | - | - | - | - | - |
| 4. Did the authors use a sustainability framework? If they created their own, is it clear and is it clear how the various components interact? | No | Yes | Yes | Yes | Yes | - | - | - | - | - | - |
| 5. Are the results transferable? | No | Yes | Yes | Yes | Yes | - | - | - | - | - | - |
| 6. It is clear how the content could be used? | No | Yes | Yes | Yes | Yes | - | - | - | - | - | - |
| **Total Score** | **3** | **6** | **6** | **6** | **6** | **0** | **0** | **0** | **0** | **0** | **0** |
| **Rank** | **Moderate** | **Strong** | **Strong** | **Strong** | **Strong** | **Weak** | **Weak** | **Weak** | **Weak** | **Weak** | **Weak** |

**Note: 0-2=weak, 3-4=moderate, 4-5=strong**
