# Supplementary material for: Sustainable by design: a systematic review of factors for health promotion program sustainability
Source: BMC Public Health. 2020 Jun 19;20:964. doi: 10.1186/s12889-020-09091-9 (PMC7304137; doi:10.1186/s12889-020-09091-9)
Supplement: Supplementary file 3 — Additional file 3. Characteristics of included papers. [file 12889_2020_9091_MOESM3_ESM.docx]

**Additional File 3. Characteristics of Included Papers**

| **Study** | **Design** | **Purpose of the study** | **Definition of Sustainability** | **Sustainability Framework Used/developed** | **Barriers** | **Facilitators** |
| --- | --- | --- | --- | --- | --- | --- |
| Altarum Institute, 2009 | Literature Review | To understand how to define, conceptualize, and measure sustainability across health programs and initiatives | Shediac-Rizkallah and Bone, 1998  1. Individual level: maintaining health benefits for individuals after initial program funding ends, particularly continuing to achieve beneficial outcomes for new clients.  2. Organizational Level: continuing program activities within an organizational structure and ensuring that program goals, objectives, and approaches adapt to changing needs over time.  3. Community level: building the capacity of the community to develop and deliver program activities, particularly when the program worked via a community coalition or other community capacity-developing process. | Conceptual Model for Evaluating the Sustainability of Community Health Initiatives (Beery et al., 2005)  Examines three key initiatives (partnership/organizing entity, activities, and other community capacity building) and the transition activities which sustain them:   - identifying most effective programs to be sustained; - finding resources; - locating new homes for programs/services; - creating new staffing models; - defining a role for partnerships; - ensuring ongoing implementation and enforcement of policy changes; - ensuring durability of systems changes; - creating systems for long term evaluation and monitoring   Three intermediate (sustainability) outcomes are identified:   1. Sustainability of the partnership 2. Sustainability of activities 3. Sustainability of other community capacities   Long term outcomes are health outcomes.  Influencing factors, within funder, organization and broader community environments, affect all stages in the model. | - Availability of resources - Finding new funding - Political climate - Limited access to resources to support post-funding sustainability evaluations - Changing health needs of communities | - Marketing strategies to increase visibility of the program and attract new audiences - Managing and leveraging resources such as management of finances and sound accounting practices - Assess organization's capacity to sustain the program: examine the mission statement, board/governance, program management, financial management, planning and human resources management - Fosters ownership of programs and system changes by organization staff and community partners - Performance tracking - Program that is based in a theory of change organizational theory, and financial theory - Engage in purposeful strategic planning for sustainability at inception and throughout lifecycle - Strategic development: use results of self-assessment to develop a strategic sustainability plan - Recruitment of champions to advocate publicly for the program - Diverse funding sources - Self-assessment - Proper implementation - Cost effective program design - Alignment with client needs and host organization mandate |
| Carstensen et al. 2019 | Qualitative multiple case design | To characterize the extent of sustainment and identify factors that influenced sustainment in four community mental health organizations. | Scheirer, 2013  Sustainment is the continuation of an intervention's components and activities after an initial period of funding ends or following the initial implementation | Normalization Process Theory (NPT) (May and Finch 2009)  NPT is concerned with making practices "routine elements of everyday life" and "sustaining embedded practices in their social contexts". There are four generative mechanisms for normalisation:   1. Coherence: the process of sense-making in relation to the introduction, implementation, and possible sustainment of an intervention 2. Cognitive participation: the actual engagement of actors in the change process and the factors affecting this engagement 3. Collective action: the operationalization of the intervention in the organization and the organizational features that influence the normalisation of the intervention 4. Reflexive monitoring: the process of formal and informal evaluation, processes where actors reflect on, discuss, and try to assess the implications and impact of the intervention for themselves and its users. | - The use of designated key persons among staff to develop, run and adjust health promotion activities. This meant the key person became highly person-bound as opposed to a stable and positive in the organization. Managerial attention and prioritization of resources dedicated to the key person was limited - Perceived lack of time and staff resources to sustain the implemented health promotion activities - Maintaining continuity in planned activities with users - Complex and heterogeneous circumstances of users - Competing tasks and the complexity of needs from users | - Local policies on health promotion such as policies on healthy food - Activity plans tailored to individual users (mandatory municipal action plans for users) - High degree of coherence generated during and after implementation. Understanding of health promotion as a distinct component of treatment and as an important part of working with the personal development of users - Common understanding among staff and management that the health promotion initiatives were meaningful and contribute to broader societal goals of tackling inequalities in health. - Health promotion initiatives fit well with the mission, goals, and strategies of the organization and make sense with day-to-day practice - Health promotion initiatives are relevant to users and benefit users - High level of management commitment/engagement to sustainability and staff engagement - Development in the external political-administrative context of the provider organization (such as new national health policy agendas) which were in line with the health promotion agenda - Designating key persons among staff to develop, run and adjust health promotion activities - Ways of monitoring health promotion initiatives e.g. monthly reports, ongoing discussions about policies and activities at meetings). - Key persons regularly "took stock" of participation in activities and comments from users to adjust activities. - Reflexive monitoring related to individual users included annual health checks and mandatory municipal activity plans - Opportunities for reconfiguration of the intervention activities - Consistency in the way sustainability of staff engagement was experienced |
| Casey et al. 2009 | Qualitative Study | To investigate the factors affecting sustainability of a health promotion program within sport and recreation organizations. It focused on identifying strategies for funding agencies and other organizations to seek to promote and sustain health promotion by sport and recreation organizations | Shediac-Rizkallah and Bone, 1998  Three perspectives about the concept of sustainability:   1. Maintaining health benefits achieved through an initial program 2. Continuation of a program* 3. Building the capacity of the recipient community   *article focused on this part of the definition  Steckler and Goodman 1989  Institutionalization is the continuation of a program, or program activities within an organization beyond the funding period. | Assessing if a program is likely to be sustained checklist from “Indicators to help with Capacity Building in Health Promotion”, New South Wales Health page 29-30, Hawe, 1999  14 questions in three categories:   1. program design and implementation factors 2. factors within the organizational setting which are known to relate to the survival of a program 3. factors in the broader community environment which affect how long programs last | - Continued funding : any reduction is thought to make it impossible to sustain the program, and that it would reduce organizational capacity to deliver the HPP - Trying to find funding could also distract program staff from their immediate job and the program could suffer as a result - Due to implementing the program, there may be limited capacity to access new revenue and limited capacity to generate new resources | - Stakeholders are aware of the program and involved in its development - A network of organizations that would advocate for the HPP if funding was threatened. These networks also improved cross sectoral partnerships between various sectors who have identified the HPP focus as a factor for poor health outcomes - Formal and informal training of staff in the program - Workforce development opportunities provide staff with new skills - Program champions, influential individuals within the organization that can act as advocates - Health promotion program scheme complements other Regional Sports Assembly (RSA) funded programs and was integrated within existing programs - Alignment with community opinion and primary care partnerships - The RSA is aligned with the organizations values and focus areas |
| Elsworth and Astbury 2005 | Mixed methods | To present a model of sustainability of pilot projects in health promotion and propose enabling strategies through which sustainable processes and outcomes are achieved. | A sustainable program is one that has become routinized in an organization as well as standardized within policy making institutions. | Multi-Level Model of Project Sustainability  Examines context (institutional, social and organizational) and list four enabling/constraining conditions:   - Conceptual model - Affordance - Support - Constraints   Lists mechanisms for sustainability and outcomes at the institution, organization/client and project level. | - Constraints from formal and informal institutional policies, guidelines and rules - Lack of a clear conceptual framework from the outset of the project - Local and administrative tasks, such as parking, food service regulations, opening hours and maintenance of equipment | - Use of a conceptual model, either 'external', (e.g., from policy documents) or project developed logic model, program theory etc. - Affordance: the policy or program provides opportunity for local innovation - Support from internal project champions, external advisors and networks |
| Garst et al. 2017 | Empirical qualitative study | To develop a framework for sustainability for health care interventions including 1) sustainability outcomes and 2) barriers and enablers for these outcomes | Scheirer and Dearing, 2011  The continued use of program components and activities for the continued achievement of desirable program and population outcomes. | Framework for Sustainability of Translational Research Projects (adapted from Scheirer 2011)   1. Sustainability at intervention sites  - Maintenance of benefits for participants and community - Maintenance of intervention activities and policies - Maintenance of community-level partnerships - Sustained attention to issue and intervention in community  1. Diffusion to wider community  - Diffusion of intervention activities and policies - Attainment and maintenance of high-level partnerships - Diffusion of attention to targeted issue and intervention  1. Replication at other sites  - Replication of intervention activities and policies - Attainment of new community-level partnership - Replication of attention to targeted issue and intervention | - Loss of organizational memory - Rigidity of existing health care structures and healthcare budget can prevent maintaining the complete intervention - High staff turnover - Lack of funds to conduct data analysis - Not having full project findings - Time it took for translating evidence into policy and the decision making process for policy development, as it relates to diffusion | - Continuous communication with stakeholders and policy makers - Regular communication through appropriate channels and communication to general public for increased issue severity - Local ownership: close involvement of stakeholders who will be responsible for sustaining the project - Level of communication between partner organizations - Involvement of local stakeholders was shown to support maintenance of the benefits for participants - Collaboration between original and new intervention site by involvement of the investigator of the original site in setting up new intervention site; communication between original site and new site coordinated by overarching organization (grant supplier) - Identification of required health authorities and regional and national stakeholders for support/political support - Division of tasks between staff and partners - Timing of involvement of stakeholders - Integration of intervention into regional/national health policies - Building an evidence base with relevant types of evidence in order to obtain policy maker support - Ongoing education of health professionals regarding practices related to the intervention helped to sustain the attention on the issue and intervention in the community |
| Hill et al. 2011 | Workbook | To assist agencies to plan program sustainability from the outset, thereby improving the likelihood of maintaining effective falls prevention programs longer term. | Sustainability refers to the long term continuation of effective programs, or, where there is a set of activities aimed at achieving the programs objectives that are incorporated into organizations routines. | 12 factors for sustainability and sustainability checklist   1. Establishing a need for the program 2. Core business and fit with organisation 3. Benefits (including promotion and marketing) 4. Organisational capacity 5. Planning from the outset: implementation, evaluation and sustainability 6. Program adaptability 7. Integrating program activities into existing routines 8. Partnerships 9. Stakeholder engagement: staff, management, partner organisations and older people 10. Capacity building: staff and partners 11. Funding and resources 12. Program champions | - Dependence on long term external funding may be detrimental to sustainability, as well as solely depending on short term funding - Insufficient funds and resources - Lack of management support - Availability of qualified staff to operate program undermines implementation and long term sustainability of the program - Organizational capacity: lack of resources, staffing and leadership - Stakeholder engagement: a program cannot be sustained if the people responsible for running, supporting or using the program do not see its value - Integrating programs into existing routines-informal organizational operational systems - Champions: losing key staff, particularly champions, not having a proper handover period when the champion leaves - Core business/fit with organization-conflicts with fundamental beliefs, norms and values can undermine the program and the processes put in place to integrate the program | - Clear benefits and active promotion of the program - Sufficient resources for the program, including time - Demonstrating the potential and actual benefits of the program. Data and evidence on the effectiveness of the program may be needed for managers, staff, partners and clients to embrace the program and sustain it. Important to have good data, and that program objectives are achievable and objectively measured - Assess the capacity to run the program in the short term and long term. Determine whether there is a need for partnerships with organizations that have the capacity to conduct the program - Forming and maintaining key networks and working relationships - Program adaptability: ability of the program to be adapted to local operating conditions and needs can make it more feasible. Being able to adapt to broader environment like the community, organisational changes, research knowledge while still maintaining critical components of the program help sustain it - Integrating program activities into existing routines, roles, processes and programs within the organization - Monitor and revisit program objectives and strategies - Plan for sustainability from the outset. Program planning, implementation and evaluation and sustainability happen together - Build capacity of staff and partners. This includes training staff and should be identified at the start of your program. Also involves professional development for existing and new staff - Credible/enthusiastic program champions to muster commitment and engagement within the host organisation and partner organisation. Staff who can embrace change are more likely to implement and integrate the program into their practice - Engage management to influence organisational priorities and programs and re-allocate funding. They are good champions. - Program champions-are important for implementation and long term sustainability, during all phases of the program (planning, implementation, and sustainability) - Core business/fit with organisation: the better the program fits with the organisations values, goals and core business the more easily the program can be incorporated into the organisation and be sustained long term. - Establishing a need for the program: a program designed and implemented to meet a local need or demand is more likely to be sustained - Implementation: the greater extent and reach of your program during implementation the more likely the program will be sustained |
| Hodge and Turner, 2016 | Literature review | To:  a) Review existing empirical literature to synthesize themes around the mechanism and methodology used to evaluate program sustainability and factors that facilitate success or create barriers to program sustainment.  b) Draw on themes to develop a sustained implementation support framework for Evidence Based practice (EBP).  c) Use this framework to propose an approach to planning and monitoring EBP implementation and sustainment. | Sustained program implementation (whether a program operated over multiple years.) | Sustained Implementation Support Framework for Evidence-Based-Programs  18 enablers/barriers in 3 categories:   1. innovation characteristics; 2. capacity factors within the workplace; 3. process and interaction factors (internal and external) | - Lack of resources such as transportation and essential materials and uneven support for program training - Lack of financial resources and support - Lack of fiscal oversight of program expenditures - Lack of commitment and team work when it comes to integrating the new program - Availability of qualified staff and ability to attract and retain them due to funding restrictions, often requiring the use of less qualified staff - High staff turnover - Lack of cohesion in the workplace (organization) - Workplaces with weak support functions for staff - Lack of feedback from superiors - Organizations with weak support functions - Lack of engagement effort and rapid geographical spread of program activity with little time for engagement - Lack of political support - Decreases in planning activity - External partners did not support the agency’s effort to implement the program - Time and implementation burden experienced by providers - The program is not viewed as a regular part of service delivery or if it is difficult to balance program and work commitments - Practitioner attitude with regard to openness to the program could also be a facilitator - Abandonment of team meetings and indicators - When programs envision large scale implementation simply as a training cascade (i.e., lack of a pilot phase) | - Reporting program outcomes to a large audience - The development of effective plans to secure funding and sustain program activity. Plan for 10 year lifespan. Plan early to address sustainability from the outset - A positive workplace climate is necessary throughout the implementation process to make effective decisions and take action to enhance program and practice sustainment, good superior involvement and peer support are needed - A positive workplace climate and teamwork as a core value can improve sustainment - Supportive work environment-pragmatic support for staff - Staff awareness of workplace values, mission and goals and program congruence and integration with those values - Involving all staff and community members or community systems in new programs - The extent of a leaders commitment to the workplace mission - Leadership style: transformational senior leadership which articulates values and vision that motivate and inspire others in a way that achieves cohesion, sets priorities, promotes learning and models quality - Supervision and peer support - Monetary incentives to improve retention of staff - Systems that ensure access to ongoing support and supervision leads to better sustainment - Community ownership i.e., involvement of village leader - Engage the end user and involve stakeholders throughout the entire process using a participatory approach to program planning - Sustainment is more likely when key partners firmly commit to support adoption but also long term effort of continuously ensuring programs perform solidly - Collaborative partnerships: involve stakeholders who contribute to and gain from partnership to reduce the deterioration of the capacity built. Partnerships need to be high functioning and involve stakeholders. Gain support of elders in indigenous communities, when partnership members are also training in the program itself - Program benefits and burden: provider perception of program characteristics, programs that are appealing, easy to implement and visibly effective; practitioners attitude with regard to openness to the program - Stability of the socio-economic and political environment - Political support - Ability of program to be adapted: adjustment made to mirror local circumstances - Funding and policy are broad constructs that represent the degree to which the program was supported by parties outside the community - Integrating data collection and scoring applications into a service delivery system - The degree to which program effectiveness and implementation outcomes are clearly monitored, acted upon and fed back to staff and the alignment of that feedback with program delivery expectations - Reporting program outcomes to a large audience, integrating data collection and scoring applications into a service delivery system; ongoing measurement of indicators of sustainment and the evaluation of quality of services - Integration of the program: the extent of a leaders commitment to the workplace mission so it aligns with staff awareness of workplace values, mission and goals and program congruence and integration with those values appear to important for sustainability - Sustainment planning: the degree to which professionals and individual work places have developed effective plans to secure funding and sustain program activity are important correlates of sustainment, should be planned for 10 year lifespan, best done early in the planning stage - Having a handover plan - Practitioners attitude with regard to openness to the program - Technical assistance and ongoing support: level and timing of support dedicated to implementation is associated with sustainment - Professional training systems that promote active skills training are good for practitioner self-efficacy; perceived competency in program skills - Key program champion: someone who advocates for the program or approach by providing support through the duration of the project - Fit between program and the values, beliefs and needs of the provider, better sustainment if they fill a gap in the health system, programs that are easy to implement and visibly effective - Program familiarity and competency: the degree of complexity or difficulty in understanding and implementing an EBP; previous experience implementing a program, clear understanding of logic model - Involving all staff and community members or community systems in participatory planning - Implementing the program with practitioner feedback |
| Office of Adolescent Health, 2014 | Resource guide | To provide direction for creating a sustainability plan specifically for programs and services aimed at improving adolescent health. | Effectively leveraging partnerships and resources to continue programs, services and or strategic activities that result in improvements in the health and wellbeing of adolescents.  OAH recommends that grantees create their own definition of sustainability. | OAH Framework for Program Sustainability   1. strategize (create an action strategy) 2. assess the environment 3. lead (identify, engage and develop leaders) 4. evolve (remain flexible and evolve) 5. communicate (with stakeholders) 6. Integrate (integrate program services into community Infrastructure). 7. partner (build strategic partnerships and mobilize the community) 8. diversify (secure diverse financial opportunities) | - Relying on a single source of funding - Securing funding - Achieving local buy in - Creating and maintaining partnerships - Securing local political support - Providing services to special populations - Strategic planning and prioritizing sustainability planning | - Create an action strategy: plan early, consider sustainability before applying to funding, create a shared vision of sustainability with partners and community leaders, revisit sustainability plan often, identify who is responsible for carrying it out, measure service success, identify appropriate indicators of success and develop a process for collecting data, measuring progress and incorporating changes based on data - Assess the environment: assess internal and external environments, consider organizational, financial and policy environments at the local, state and national levels, embed continuous assessments throughout the life of the program or service, use the information to decide what should be sustained, conduct a situational assessment - Identify, engage and develop leaders: identify strong internal leaders, keep organizational and senior leaders engaged through strategic updates, identify external community champions, promote leadership development and shared leadership opportunities - Remain flexible and evolve: match services to community needs and reframe work to new priorities, plan in advance for staff and leadership changes, be innovative and find new opportunities, be prepared for them, speak with others to learn about trends - Communicate with stakeholders: formulate a communication approach and messages, collect and share stories regularly with stakeholders, create an online presence, encourage program leaders, strategic partners and community champions to share - Integrate programs and services into local infrastructure: determine which program components could be integrated into other settings, streamline service delivery, policies and practices, integrate programs, services, and practices into the broader community fabric - Create strategic partnerships and mobilize the community: community mobilization can bring together partners across different sectors to advocate for your programs goals, support service delivery and advance sustainability efforts. Strategic partnerships can increase program and service capacity, increase awareness of services, maximize visibility of existing resources and decrease the likelihood of duplicated efforts. It is important to have a shared vision as this can help secure diverse funding, leverage communication strategies to build public trust, and advance policies that support the program. Build sustainability directly into your partnership agreements. Partnerships should advance a common goal and help with long term program sustainability - Having diversity of funding sources contributes significantly to sustainability. Consider making certain program components fee for service, create a specific place in the budget for sustainability, and build grant writing capacity in staff. |
| Office of Adolescent Health, 2017 | Framework | To provide a framework for sustainability which can be used to ensure continuity of a program and institutionalization. |  |  | - Leaders may be a barrier to sustainability, usually when there is just one (as opposed to having a leadership team) |  |
| Paine-Andrews et al. 2000 | Empirical study | To conduct an empirical study of the strategies used to promote sustainability of community health initiatives. | The extent to which community changes facilitated by the initiatives remained in place after grant termination and the extent to which the initiatives themselves remained in place after grant termination. | Model for Institutionalizing Health Promotion Programs (Steckler and Goodman, 1989)  Six practice implications:   1. Cultivate a program champion 2. Favor organizations with mature subsystems 3. Favor organizations in which health promotion “fits” with the organization’s mission 4. Avoid brokering relationships 5. Alter lengths of funding periods 6. Fund existing worthy programs |  | - The vision and commitment by project leadership both paid and unpaid. Initiatives with directors, staff and key partners or board members that were strongly committed to building healthy communities for young people and saw the need for facilitating community change tended to work harder to sustain and support the initiatives - Level of initial commitment of the lead agency to the project: those initiatives in which the lead agency remained actively involved in the project meetings helped connect project staff with key local leaders and the commitment of the lead agency to the mission of the initiative - Strength of alliances with organizations with similar missions - Success of community changes from the perspective of the community - The type or attributes of community changes facilitated by the initiative - The more closely a community change addressed a perceived need (from the community perspective) the more likely the change was sustained - Support from the lead agency and associated leadership within the agency - Partnerships with health departments - The extent of which volunteers championed community changes |
| Sadof et al. 2006 | Mixed methods | To describe qualitative and quantitative aspects of each [Inner-City Asthma Intervention] site to identify strategies that were successful in sustaining sites after funding had ended. | Scheirer, 2005   1. continuing to deliver beneficial services (outcomes) to clients (individual level); 2. maintaining the program and/or its activities in an identifiable form (organizational level); and 3. maintaining the capacity of a community to deliver program activities after the initial program created a community coalition/structure (community level)   For this article, sustainability is defined as the continuation of the central elements of the Inner City Asthma Intervention program. | Framework for Conceptualizing Program Sustainability, Shediac-Rizkallah and Bone, 1998  Examines factors at three levels:   1. Project design and implementation factors 2. Factors in the broader community environment 3. Factors within the organizational setting | - Recruiting and retaining staff | - Local media coverage - Community had had public awareness campaign on asthma prior to program being implemented - Prior experience with an asthma program - Having multiple funders - Staff members are responsible for developing community linkages - Community linkages and data collection appear to have the strongest association with sustainability - Working in conjunction with partners, community organizations, clinics and providers at the local level (an indicator for community involvement is the number of community partners) - Physicians and funders working to bring evidence-based interventions into the community may best achieve sustainability by partnering with community stakeholders and local institutions - Patient symptom and health utilization data were gathered and connections were established among the institution, local funders and community members - Being able to collect the necessary data to communicate success in a tangible fashion to the organization - Implementation design, which incorporated the collection of outcomes data may help to encourage potential funders to sustain effective program - Type of data collected (symptom data, health service encounters), presenting data to future funders - Sufficient funding is thought to allow programs to collect data on health outcomes - Data collection and analysis - Evaluation is embedded in the program from the start - Program advocate/champion - Community service recognition - Local politician support |
| Scheirer, 2005 | Systematic review | To examine the types and extent of sustainability achieved for the programs studied [in the systematic review] as well as summarizing the findings concerning factors that were found to contribute to greater likelihood of sustainability | Shediac-Reizkellah and Bone, 1998  Three definitional measures of sustainability:   1. continuing to deliver beneficial services (outcomes) to clients (individual level); 2. maintaining the program and/or its activities in an identifiable form (organizational level); 3. maintaining the capacity of a community to deliver program activities after the initial program created a community coalition/structure (community level) | Framework for Conceptualizing Program Sustainability, Shediac-Rizkallah and Bone, 1998  Examines factors at three levels:   1. Project design and implementation factors 2. Factors in the broader community environment 3. Factors within the organizational setting | - Staff turnover - Medical practice ownership changes - Silo structures as they did not allow infusion of multidisciplinary influences from the community - Deciding to continue the program before examining evaluation data - Frequent changes to meet the priorities of new funders could lead to loss of focus and less sustainability - Low level of implementation early in the project | - Funding from other sources, particularly the availability of a large number of funding sources or the transfer of support to local governmental sources - Multiple strategies used for obtaining funds - Having a pair of coordinators to staff the program (rather than one coordinator) - Existing organizational capacity - Support from other organizations in-kind, such as expert advice in fundraising, political support or to help mobilize clients to advocate for new funding - Support from other stakeholders in the community - The use of volunteers or low cost ways of delivering services - Benefits to staff members and or clients that are readily perceived - Program staff members’ perceptions of the program benefits even if not confirmed by evaluation - The program itself is modifiable over time - Leadership and staffing - Extent to which the program can be modified to adapt to the organization (fit) - Fit of the new program within existing organizational mission and/or standard operating procedures - Substantial fit with the underlying organizations missions and procedures - Programs that were modifiable at the local level as it was easier to make changes in the initial program design to address greater perceived need, or make it easier to deliver locally - Use of evaluation data for gaining support needed for continuation - Early planning of sustainability - External technical assistance from program developers or funders - Having a program champion, sometimes the executive director, who can advocate for the needs of the program and help secure resources for its continuation - Project activities that could be sold as contributing to the organizations were more likely receive support and therefore be sustained - Project activities that could readily fit into existing tasks and procedures were more likely to receive operational support - Continuous staff discussion about how to implement and sustain the program |
| Schell et al. 2013 | Mixed methods (literature review and concept mapping) | To present a new conceptual framework for program sustainability capacity for public health programs. | The ability to maintain programming and its benefits over time.  Sustainability capacity is defined as the existence of structures and processes that allow a program to leverage resources to effectively implement and maintain evidence based policies and activities. | Framework of public health program capacity for sustainability:   1. funding stability 2. political support 3. partnerships 4. organizational capacity 5. program adaption 6. program evaluation 7. communications 8. public health impacts 9. strategic planning |  | - Stable funding - Political support - Partnerships - Organizational capacity - Program adaption - Program evaluation - Communications - Public health impacts - Strategic planning |
| Shediac-Rizkallah and Bone, 1998 | Literature review | To synthesize a diverse literature on what is known on sustainability in order to determine indicators for sustainability over time; and to present potential guidelines and strategies for fostering program sustainability within the dynamic context of community. | Sustainability is a global term used to refer to the general phenomenon of program continuation. There are three perspectives on sustainability:   1. maintain health benefits achieved through the initial program 2. continuation of the program activities within an organization structure 3. building the capacity of the recipient community | Framework for Conceptualizing Program Sustainability, Shediac-Rizkallah and Bone, 1998  Examines factors at three levels:   1. Project design and implementation factors 2. Factors in the broader community environment 3. Factors within the organizational setting | - Short grant periods for establishing new programs - Integration with existing programs/services: 'vertical' or standalone self-contained programs as they are more vulnerable and create internal jealousy - Preventative programs are harder to sustain than curative programs - Projects imposed by a funding agency are less likely to be sustained - Competing problems such as poverty, unemployment, crime may stop a program from continuing - Short term horizons of government and funding agencies and short budget cycles and internal political pressures - Less favourable environment for sustainability due to greater economic deterioration and weaker governmental institutions e.g., Africa versus Central America - Excessive outside funds as the program exceeds local resources | - Project financing is likely the most important factor. Careful planning by donors and grantees for eventual cutbacks in funding, the ability to identify costs and set realistic fees, adopting an entrepreneurial spirit in seeking alternative sources of funding and diversification of services - Institutions that are well integrated have goal structures that are consistent with the project goals and have strong leadership, are stable and mature, and experienced with program implementation. Integration with existing programs/services: programs that are well integrated with existing systems or into standard operating procedures of the host organization - Sense of ownership: increased partnership increased community capacity to sustain the program - Objective evaluation evidence that program is worth sustaining - Participatory approach: projects imposed by mutually respectful negotiating process, providing services that are in need by the community, negotiation of consensus building process to reach compromises with community members - Project duration: aim for five years - Curative programs are easier to sustain than preventative projects. - A broad range of health professionals, health institutions, community groups and private citizens are involved in a “collective attack” on health risk behaviours and the conditions that produce or support them - Professional and nonprofessional training, those trained can continue to provide benefits - Program champions/leadership: influential individuals within the implementing organization acting as program advocates or champions. Champions with mid-upper level managerial positions, with a sense for the compromises necessary to build support for the program and negotiating may be best for promoting sustainability. Endorsement and support of the program from the top of the host organization |
| Whelan et al. 2018 | Systematic narrative analysis | To identify factors considered fundamental to the sustainability of obesity prevention interventions and their effects and to overcome challenges in sustainability; and to determine:   - how the term sustainability is used in obesity prevention interventions; - what factors are related to the sustainability of obesity prevention interventions and their intended outcomes | Sustainability of obesity prevention is defined as changes in behavioural determinants and/or BMI at least 12 months post the initial impact has been demonstrated. | Ten Key Elements of Sustainability (Whelan, Love and Pettman 2014)  Ten key elements of sustainability:   1. planning for sustainability early in implementation phase 2. using evidence to identify the issues and potential interventions 3. commitment and support from host and stakeholder organizations 4. engagement with community and strategic partners 5. champions 6. capacity-building, community and organizational capacity 7. evaluation 8. policy 9. evolve and adapt 10. funding from diverse sources | - No availability of local staff for the health promotion program - Staff turnover - Unclear governance structures - Activities that counter the purpose of the intervention, for example unhealthy fundraising options undermined sustainability | - Communication with target population: multiple messaging strategies such as websites, e-communication and newsletters. Social marketing is important for reaching all demographic groups. Communication helped sustainability when the emphasis was placed on how, what and with whom to share information early in the intervention; ongoing communication and repeated messaging; having a formalized communications plan and ensuring consistent messaging - Dedicated funding for sustainability, actively seeking out grant funding, or independently generating funding to achieve a sustainable outcome. Being able to replicate the intervention at a reasonable cost - Governance: steering committee, having a memorandum of understanding and appropriate documentation, project management committees, or inclusion of community in governance structures - Interventions that were able to mobilize existing resources (financial and human resources) enhanced sustainability - Human resources related to sustainability through employment of the local workforce or the creation of new positions for local people because it built local capacity, but important not to overload local workforces - Community engagement such as broad community consultation and collaboration, and understanding public 'sentiment', with buy-in from everyone. Mobilization of community wide efforts was directly linked to sustainability, and when policy interventions were supported by an engaged community interventions were strengthened. Having the community be part of the intervention design through a participatory approach helped - Multidisciplinary teams may be required. Partner with parents, teachers and children along with community organizations. Partnerships with existing organizations, industry and local business was also important - Partnerships that lead to sustainability were thought to enhance the program by providing tangibles resources to the intervention, and were effective when there was regular communication between partners and links to existing community resources and organizations - Partnerships were effective for program sustainability, sometime first partnering with those who are eager to participate which resulted longer lasting support - Adaptation: implementation, intervention flexibility and adaptability to local contexts, implementation fidelity refers to cultural adaptation and flexibility of content and delivery to facilitate real time problem solving - Involvement of stakeholders in the design, implementation and evaluation of program - Including sustainability as a separate measure outcome in evaluation - Workforce development such as skill development related to the program content and intervention delivery. Low-cost delivery methods for training were suitable to achieve this - Ongoing capacity building of the partners is needed to sustain the partnerships - Identifying a champion who is from within the intervention, part of management, responsible for identifying training needs, or a role model. Leaders either formal or informal were linked to sustainability of the program - Developing policy as an intervention strategy with the stated purpose of sustaining the program. |
| Wisener et al. 2017 | Collective case study | To Identify the factors that promote and/or inhibit sustainable health education initiatives and integrate these factors into a set of sustainability tools that can be used to support effective program development in Aboriginal communities. Also, to examine three case studies (Community Learning Centre-CLC) in order to understand 1) how to transition from funding to post-funding 2) whether or not the transition was successful 3) what factors positively or negatively influenced success. | A sustainable program is   1. receptive to change and adaptable 2. an innovative strategy that provides continued benefit 3. fully integrated into normal operations post-project funding 4. of benefit to diverse stakeholders | Sustainability framework based on the seven factors revealed in the study.  Seven factors that promote or inhibit the sustainability of each CLC:   1. Community uptake-if and how the users access the program, including the diversity of users, the frequency with which they access the program and what they use the program for 2. Environment-the program setting. Also addresses competing community resources, community politics and the effect of similar programs accessible in nearby urban/non community settings 3. Stakeholder support-the presence and extent of program support exhibited by various groups, including but not limited to community members; band administration; band council; national council; program staff; and academic institutions 4. Presence of a champion-the most positive form of a stakeholder support exhibited by individuals either directly or peripherally involved in the program. Effective champions possess leadership skills 5. Funding-the communities ability and motivation to identify and allocate funds to support the programs ongoing operation 6. Fit and flexibility-the extent to which the program is in line with community priorities as a whole and how adaptable it is in meeting individual user needs 7. Capacity and capacity building-the community’s general capacity/ability to sustain the program, the capacity with program staff to champion the program, and whether the program increased community capacity to address other issues. | - The communities’ ability and motivation to identify and allocate funds to support the programs. Could be an excuse to not sustain a program. If not enough stakeholders invested in the group then there would not be enough effort to secure funding [factors that promote or inhibit sustainability]. - Stakeholder awareness of and support towards the learning centres corresponded to increased utilization of the program [factors that promote or inhibit sustainability]. - Uptake by the community-if and how users access the CLC, data on this would be useful [factors that promote or inhibit sustainability]. - Capacity and capacity building emerges as a complex indicator of sustainability, building capacity around community skills, structures, resources and commitment helped to sustain program impacts [factors that promote or inhibit sustainability]. - Perception of a champion-championing appeared to increase success of the program even compared to stakeholder groups support, having one key individual was more beneficial [factors that promote or inhibit sustainability]. - Fit and flexibility in meeting community priorities-whether the program is in line with communities priorities as a whole, and whether the community defined health education issues drove the programs resource development [factors that promote or inhibit sustainability]. - Fit with individual community needs- whether the program has the ability to meet individual needs [factors that promote or inhibit sustainability]. - Location, infrastructure, staffing, technology/equipment, and other CLC specific factors that influenced CLC use and sustainability [factors that promote or inhibit sustainability]. - Excessive outside funds as the program exceeds local resources [factors that promote or inhibit sustainability]. | - The communities’ ability and motivation to identify and allocate funds to support the programs. Could be an excuse to not sustain a program. If not enough stakeholders invested in the group then there would not be enough effort to secure funding. - Uptake by the community: data on if and how users access the CLCs - Stakeholder awareness and support towards the learning centres corresponded to increased utilization of the program - Capacity and capacity building emerges as a complex indicator of sustainability. Building capacity around community skills, structures, resources and commitment helped to sustain program impacts - If health outcomes could be continually realized with community based resources instead of external funding then some capacity building had taken place. Three main contexts: the communities’ capacity to sustain the CLC; technical leads to champion the CLC; and the communities increased capacity to address other health education issues in the community - Having a champion appeared to increase success of the program even compared to stakeholder groups support. Having one key individual was more beneficial - Fit and flexibility in meeting community priorities: whether the program is in line with communities priorities as a whole, and whether the community defined the health education issues that drove the programs’ resource development. Fit with individual needs- whether the program has the ability to meet individual needs - Program factors such as location, infrastructure, staffing, technology/equipment, and other CLC specific factors that influenced CLC use and sustainability |
