# Supplementary material for: Sustainable by design: a systematic review of factors for health promotion program sustainability
Source: BMC Public Health. 2020 Jun 19;20:964. doi: 10.1186/s12889-020-09091-9 (PMC7304137; doi:10.1186/s12889-020-09091-9)
Supplement: Supplementary file 4 — Additional file 4. Weighting of factors for sustainability by frequency, importance and relevance. [file 12889_2020_9091_MOESM4_ESM.docx]

**Additional File Four: Weighting Table for Frequency, Importance and Relevance**

| **Factors** | **Sub-factors** | **Frequency:** the number of times barriers/facilitators appeared in the extracted results | **Importance:** the number of times barriers/ facilitators were cited as particularly important to the sustainability of the HPP | **Relevance:** the number of times barriers/ facilitators were cited in Public Health Ontario’s 6-step health promotion program planning model |
| --- | --- | --- | --- | --- |
| Organizational Capacity | environment | 3 | 3 |  |
|  | fiscal oversight | 2 |  |  |
|  | governance | 2 |  |  |
|  | leadership | 6 | 2 |  |
|  | mobilize resources | 2 | 2 | 1 |
|  | organizational commitment | 5 | 2 |  |
|  | staffing | 11 |  | 1 |
|  | organizational memory | 1 |  |  |
|  | management support | 3 | 1 |  |
|  | organizational policies | 1 |  |  |
|  | organizational structure | 3 |  |  |
| **Total citations*** |  | **54** | **14** | **3** |
| Partnerships | community engagement | 3 | 3 |  |
|  | Stakeholder engagement | 4 | 1 |  |
|  | fit | 1 |  |  |
|  | ownership of stakeholders | 3 | 1 |  |
|  | participatory planning | 1 |  | 1 |
|  | staff responsible for partnership | 1 |  |  |
|  | stakeholder awareness | 2 |  |  |
|  | stakeholder commitment | 1 |  |  |
|  | stakeholder involvement | 2 | 1 | 2 |
|  | stakeholder support | 1 | 1 | 1 |
|  | community uptake | 1 |  |  |
| **Total citations*** |  | **43** | **10** | **13** |
| Strategic Planning | Assessment of environment | 1 | 1 | 1 |
|  | framework | 3 |  | 1 |
|  | integration | 6 | 1 |  |
|  | participatory planning | 1 |  | 1 |
|  | program duration | 1 |  |  |
|  | program type | 3 |  |  |
|  | sustainability planning | 5 |  |  |
| **Total citations*** |  | **27** | **6** | **7** |
| Funding | funding for sustainability | 1 |  |  |
|  | multiple funders | 5 |  |  |
|  | multiple strategies | 1 |  |  |
|  | participatory budgeting | 2 |  |  |
|  | sustainability | 2 |  |  |
| **Total citations*** |  | **23** | **5** | **1** |
| Fit/alignment |  | **22** | **6** |  |
| Evaluation | data collection | 6 | 1 |  |
|  | effectiveness | 4 |  |  |
|  | indicator of community support | 1 |  |  |
|  | involve stakeholders | 1 |  |  |
|  | resources | 3 |  |  |
|  | sustainability evaluation | 1 |  |  |
|  | use of evaluation results | 1 |  |  |
|  | monitoring | 1 |  |  |
| **Total citations*** |  | **20** | **1** | **4** |
| Capacity building |  | **17** | **2** |  |
| Champion |  | **15** | **3** |  |
| Communications | awareness | 1 |  |  |
|  | community | 1 |  |  |
|  | media coverage | 1 |  |  |
|  | promotion/marketing | 3 |  |  |
|  | results | 1 |  |  |
|  | stakeholders | 3 | 2 |  |
| **Total citations*** |  | **10** | **2** |  |
| Program implementation |  | **8** | **0** | **2** |
| Political Support |  | **7** |  |  |
| Program Adaptation |  | **7** | **4** |  |
| Public Health Impacts | perception of benefits | 3 | 1 |  |
| **Total citations*** |  | **6** | **3** | **2** |
| Socio-economic/political factors |  | **5** | **1** |  |
| Program access factors |  | **3** |  |  |
| Funder priorities |  | **3** | **0** |  |
| Policy |  | **2** | **1** |  |
| Affordance |  | **1** |  |  |
| Tailored activity plans for individual clients |  | **1** | **1** |  |

***Number of times cited in total. Not all citations were related to specific sub-factors therefore sub-factor citations do not add up to the total citations.**
